# Origin and genetic analysis of stem rust resistance in wheat line Tr129

**DOI:** 10.1038/s41598-022-08681-4

**Published:** 2022-03-17

**Authors:** Jyoti Saini Sharma, Thomas G. Fetch, Habibollah Ghazvini, Matthew N. Rouse, Tatiana Danilova, Bernd Friebe, Colin W. Hiebert

**Affiliations:** 1 Agriculture and Agri-Food Canada, Morden Research and Development Centre, 101 Route 100, Morden, MB R6M 1Y5 Canada; 2 Cereal Research Department, Seed and Plant Improvement Institute, Agricultural Research, Education and Extension Organization (AREEO), Karaj, Iran; 3 USDA-ARS, Cereal Disease Laboratory and Department of Plant Pathology, University of Minnesota, St. Paul, MN 55108 USA; 4 Department of Plant Sciences, North Dakota State University, Fargo, ND 58108 USA; 5 Department of Plant Pathology, Kansas State University, Manhattan, KS 66506 USA

**Keywords:** Plant breeding, Plant genetics

## Abstract

Wheat line Tr129 is resistant to stem rust, caused by *Puccinia graminis* f. sp*. tritici* (*Pgt*). The resistance in Tr129 was reportedly derived from *Aegilops triuncialis*, but the origin and genetics of resistance have not been confirmed. Here, genomic in situ hybridization (GISH) showed that no *Ae. triuncialis* chromatin was present in Tr129. Genetic and phenotypic analysis was conducted on F_2_ and DH populations from the cross RL6071/Tr129. Seedlings were tested with six *Pgt* races and were genotyped using an Illumina iSelect 90 K SNP array and kompetitive allele specific PCR (KASP) markers. Mapping and phenotyping showed that Tr129 carried four stem rust resistance (Sr) genes on chromosome arms 2BL (*Sr9b*), 4AL (*Sr7b*), 6AS (*Sr8a*), and 6DS (*SrTr129*). *SrTr129* co-segregated with markers for *SrCad*, however Tr129 has a unique haplotype suggesting the resistance could be new. Analysis of a RL6071/Peace population revealed that like *SrTr129*, *SrCad* is ineffective against three North American races. This new understanding of *SrCad* will guide its use in breeding. Tr129 and the DNA markers reported here are useful resources for improving stem rust resistance in cultivars.

## Introduction

Stem rust, caused by the fungal pathogen *Puccinia graminis* Pers.:Pers. f. sp*. tritici* Eriks & E. Henn (*Pgt*), poses a threat for wheat production. While resistance to stem rust had been stable in wheat cultivars for many years, *Pgt* race TTKSK (Ug99) with virulence on many genes was detected in Uganda in 1998^1^. The Ug99 race group (TTKSK and 13 variants) and some non-Ug99 races such as TRTTF, TTTTF, and TKTTF continue to spread across different countries and carry virulence to widely deployed stem rust resistance (Sr) genes^1–7^. To mitigate these threats at global and local levels, it is important to genetically characterize known resistance resources while also searching for new resistance. Globally, scientists have been exploring the gene pools of wheat and its wild ancestors to identify novel and effective resistance resources against stem rust.

Wheat line Tr129 carries resistance to both leaf rust and stem rust^8,9^. This line was reportedly developed by hybridizing the hexaploid common wheat cultivar Marquis (2*n* = 6*x* = 42, AABBDD) with the tetraploid species *Aegilops triuncialis* (2*n* = 4*x* = 28, CCUU) accession CN 34405^8^. An initial inheritance study conducted by Fetch and Zegeye (2009) found a single dominant gene in Tr129 conditioning resistance to race TPMKC. Further testing indicated the presence of two Sr genes in Tr129 that conferred resistance to *Pgt* race MCCFC^10^. Preliminary mapping suggested that these two genes were located on chromosome arms 2BL and 6AS, but the locations were not validated^11^. The objectives of this study were to determine the origin of stem rust resistance in the wheat line Tr129, determine the chromosomal locations of Sr genes present in Tr129, and identify the Sr genes as known or novel.

## Results

### GISH analysis

Genomic in situ hybridization (GISH) with probes produced from DNA of *Ae. umbellulata*, *Ae*. *caudata* or *Ae. triuncialis* did not detect *Ae. triuncialis* chromatin in Tr129. The control lines showed positive GISH signals as expected, indicating that an Sr gene in Tr129 was not derived from *Aegilops triuncialis* (2*n* = 4x = 28, CCUU) as reported by Aung & Kerber^8^ or that a translocation segment, if present, was too small to be detected by GISH (Supplementary Fig. S1). Moreover, SSR genotyping of the chromosome region flanking a resistance gene in chromosome 2B (Supplementary table S1) showed that the haplotype was more representative of Neepawa (postulated genes *Sr5*, *Sr7a*, *Sr9b*, *Sr12*, *Sr16*^12^) than Marquis (*Sr7b*). This alone indicated that the published pedigree of TR129 (Marquis*6/3/Marquis/CN 34,405//2*Marquis)^8^ was incorrect.

### Phenotyping

Wheat line Tr129 showed resistance to *Pgt* races A (TTKSK), B (RRTTF), C (QTHJF), D (TMRTF), E (TPMKC), and F (MCCFC) (Table 1) with infection types (IT) 2, 2^−^, 2^−^, 11^+^, 22^+^, and 1^−^ respectively. RL6071 was susceptible to all *Pgt* races used in the current studies with IT ranging from 3^+^ to 4. The RL6071/Tr129 F_2_ population segregated 57 resistant (R): 25 susceptible (S) plants to race A, fitting an expected 3 resistant: 1 susceptible ratio for a single dominant gene (*χ*^*2*^ = 1.31, *P* = 0.25). Segregation data for the entire (or near entire) doubled haploid (DH) population with three races (A, E, and F) allowed postulation (Table 1) of genes *Sr8a*, *Sr9b* and temporarily name *SrTR129* either singly or in combination based on avirulence/virulence attributes of each race. Although the result with race E showed a significant overabundance of susceptible lines, genetic mapping (below) confirmed that a single gene was involved. Gene *SrTr129* showed close similarity to *SrCad*, *Sr42*, and *SrTmp*^13–15^.

**Table 1:** Phenotypic ratios, chi squared analysis and *Sr* gene postulation for response to six *Puccinia graminis* races tested on the RL6071/Tr129 DH population.

Race	Code	Observed		Single gene ratio (1:1)		Two gene ratio (3:1)		*Sr* gene postulation
		Res^c^	Sus^c^	χ^*2*d^	*p*^e^	χ^*2*^	*p*	
TTKSK	A	146	122	2.15	0.14	–	–	*SrTr129*
RRTTF^a^	B	68	21	–	–	0.09	0.76	*SrTr129* + *Sr8a*
QTHJF^a^	C	70	25	–	–	0.08	0.77	*SrTr129* + *Sr7b*
QTHJF^b^	C	45	48	0.1	0.76	–	–	*Sr7b*
TMRTF^a^	D	51	44	0.52	0.47	–	–	*Sr8a*
TPMKC	E	114	151	5.17	0.02			*Sr9b*
MCCFC	F	192	67	–	–	0.10	0.75	*Sr8a* + *Sr9b*

^a^Phenotypic data for a random subset of the DH population (Subset 1).

^b^Phenotypic data for a subset of lines lacking resistance to race A (Subset 2).

^c^*Res* resistant, *Sus* susceptible.

^d^Chi-squared value.

^e^p-value for the chi-squared value.

Subsets of the DH population were selected to confirm the postulations (Table 1, Supplementary table S2). Gene *SrTr129* conferred resistance to races A, B, and C; *Sr8a* conferred resistance to F, D, and B; *Sr9b* conferred resistance to races E and F; and *Sr7b* conferred resistance to race C (Table 2).

**Table 2:** Phenotypic ratios, chi squared analysis and *Sr* gene postulation for response to four *Puccinia graminis* races tested on the RL6071/Peace DH population.

Race	Code	Observed		Single gene ratio (1:1)		*Sr* gene postulation
		Res^b^	Sus^b^	χ^*2*c^	*P*^d^	
TTKSK^a^	A	34	39	0.34	0.56	*SrCad*
TMRTF	D	37	34	0.13	0.72	*–*^e^
TPMKC	E	39	33	0.50	0.48	*Sr9b*
MCCFC	F	39	33	0.50	0.48	*Sr9b*

^a^Data from Hiebert et al. 2011^24^.

^b^*Res* resistant, *Sus* susceptible.

^c^Chi-squared value.

^d^p-value for the chi-squared value.

^e^No gene postulated for race D.

### Genotyping and mapping

**Figure 1:**
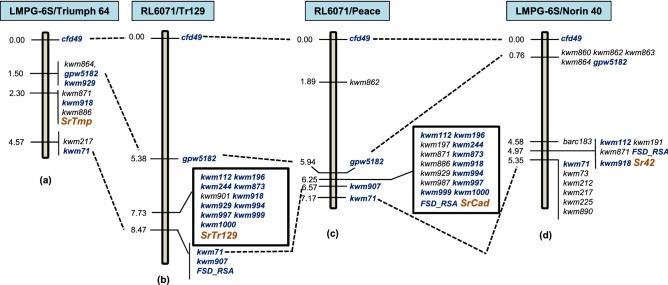
Comparison of the *SrCad* genomic region in four mapping populations: LMPG-6S/Triumph 64 (**a**), RL6071/Tr129 (**b**), RL6071/Peace (**c**), and LMPG-6S/Norin 40 (**d**). Markers shown in *dark blue font* were mapped in more than one mapping population and resistance genes are shown in *dark orange font*. Mapping distances are in centimorgans (cM).

Genotyping the F_2_ population with the 90K iSelect SNP array showed linkage between the race A resistance gene and markers on chromosome arm 6DS in the region associated with *SrCad*, *Sr42*, and *SrTmp*^13–15^. A 6DS linkage map spanning an 8.47 centimorgan (cM) genetic region was developed for the DH population using 13 KASP, two SSR, and *FSD_RSA* markers. Gene *SrTr129* conferred resistance to race A and was located to position 7.73 cM on the linkage map. It was flanked by markers *gpw5182* and *kwm71* and co-segregated with 12 KASP markers (Fig. 1), 11 of which were previously used to map *SrTmp*, *SrCad*, and *Sr42*^13,14^. Fourteen KASP markers were used to compare haplotypes in the region of the chromosome arm 6DS carrying race A resistance in wheat cultivars/lines Triumph 64 (*SrTmp*), Tr129 (*SrTr129*), Peace (*SrCad*), and Norin 40 (*Sr42*). All four haplotypes were unique, and Tr129 was differentiated from Peace, Norin 40, and Triumph 64 by three, 12, and nine SNP markers, respectively (Table 3). Previous data^14^ indicated *SrTr129* conferred resistance specificity that differed from *Sr42* and *SrTmp*, thus a comparison with *SrCad* was needed. Phenotyping the RL6071/Peace DH population revealed single gene segregation for *Pgt* races D, E, and F (Table 2). All three races virulent to *SrCad* in this population were also virulent to *SrTr129* as shown above. One Sr gene in Peace conferred resistance to races E and F, whereas independent Sr genes conferred resistance to races A and D (Supplementary table S3). *SrTr129* and *SrCad* both conferred resistance to races A, B, and C. The races used in this study detected no difference between *SrTr129* and *SrCad*.

**Table 3:** SNP marker haplotypes of wheat lines Triumph 64, Tr129, Peace, and Norin 40 with genes for stem rust resistance on chromosome arm 6DS and susceptible lines LMPG and RL6071.

	*Kwm* *112*	*Kwm* *191*	*Kwm* *196*	*Kwm* *197*	*Kwm* *244*	*Kwm* *871*	*Kwm* *873*	*Kwm* *918*	*Kwm* *929*	*Kwm* *987*	*Kwm* *994*	*Kwm* *997*	*Kwm* *999*	*Kwm* *1000*
LMPG	H	A	B	B	B	B	H	H	A	A	H	B	A	A
RL6071	H	A	B	B	B	B	H	H	B	A	H	B	A	A
Triumph 64	A	A	H	B	B	A	A	A	H	A	B	B	A	A
Tr129	A	A	H	A	A	B	B	B	H	A	A	A	B	B
Peace	A	A	A	A	A	A	B	B	H	B	A	A	B	B
Norin 40	B	B	B	B	B	B	H	H	A	A	H	B	N	N

*A* allele 1, *B* allele 2, *H* heterogeneous, *N* null.

**Figure 2:**
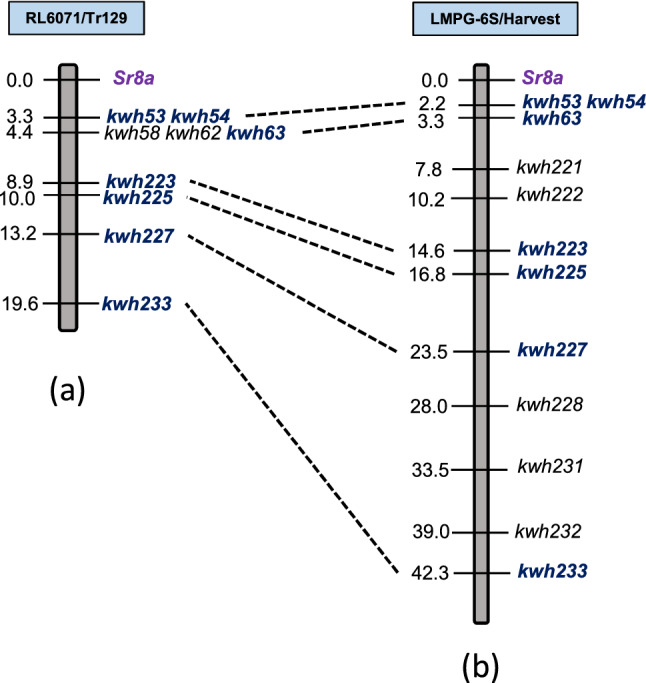
Comparison of *Sr8a* linkage maps from RL6071/Tr129 DH population from the present study (**a**) and the LMPG-6S/Harvest DH population from Hiebert et al.^16^ (**b**). Common markers between two linkage maps are in *dark blue font*, and *purple font* was used for the postulated gene. Distances between loci are in centimorgans (cM).

**Figure 3:**
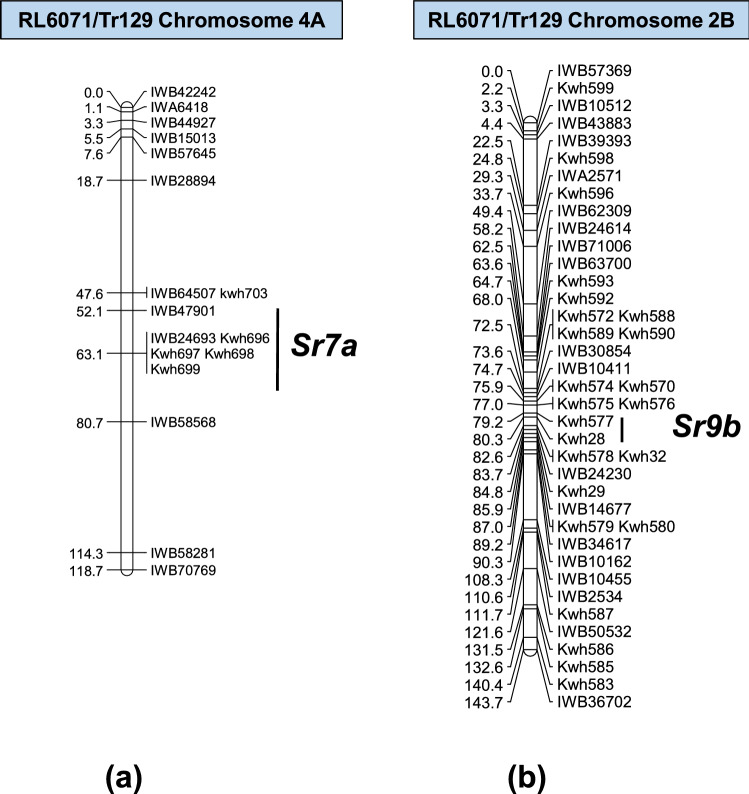
Linkage maps developed for Subset 2 DH lines. (**a**) *Sr7b* mapped at 63.1 cM on chromosome arm 4AL; (**b**) *Sr9b* mapped at 79.2 cM on chromosome arm 2BL. Kompetitive allele specific (KASP) PCR markers designated as *kwh* developed in the present study are shown on the linkage maps. Primer information is provided in supplementary table S7.

A 19.6 cM partial linkage map for chromosome arm 6AS was constructed with nine KASP markers to map the postulated *Sr8a* effective against races B, D, and F (Fig. 2). This gene was distal to KASP markers *kwm53* and *kwm54* previously reported to be closely linked with the *Sr8* locus^16^. The 90K iSelect SNP array was also used to genotype the Subset 2 DH lines that lacked *SrTr129* and identified the postulated *Sr7b* and *Sr9b* genes on chromosome arms 4AL and 2BL, respectively. The linkage map of chromosome arm 4AL located *Sr7b*, which conferred resistance only to race C. This gene was mapped to position 63.1 cM on the 4AL linkage map and was flanked by SNP markers *IWB47901* and *IWB24693*, located in the region previously associated with *Sr7*^17^ (Fig. 3a, Supplementary table S4). Phenotyping of parents and differential wheat lines carrying the *Sr7a* and *Sr7b* alleles showed that *Sr7a* was ineffective against race C (Table 4, Supplementary Fig. S2), whereas Tr129 and near-isogenic line ISr7b-Ra (CI 14,165) were resistant (IT 11 + and 22^-^, respectively). SNP marker data used to map the postulated *Sr9b* on chromosome arm 2BL that was effective against races E and F was consistent with the region known to carry *Sr9* (Fig. 3b, Supplementary table S5). The *Sr9* region has seven resistance alleles (*Sr9a*, *Sr9b*, *Sr9d*, *Sr9e*, *Sr9f.*, *Sr9g*, and *Sr9h*)^18–23^, of which *Sr9a* and *Sr9b* are effective against races E and F (Fig. 3b). Since race C is virulent for *Sr9b* and avirulent for *Sr9a*, and the Subset 2 lines lacking resistance to race A (Table 1) segregated for a single gene located on 4B, that gene is likely *Sr9b*.

**Table 4:** Stem rust reactions of parents RL6071 and Tr129, and five wheat lines carrying *Sr7a* or *Sr7b* alleles when tested with *Pgt* race C.

Line	*Sr* gene	Reaction
DK3	*Sr7a*	Susceptible
Green Na101/Mq^6^	*Sr7a*	Susceptible
EgNa101/*6 Mq 1-4-3	*Sr7a*	Susceptible
G Mq6/K117A	*Sr7a*	Susceptible
ISr7b-Ra	*Sr7b*	Resistant
RL6071	*–*^a^	Susceptible
Tr129	*Sr7b*^b^	Resistant

^a^No effective *Sr* gene present against race C.

^b^Postulated.

## Discussion

Line Tr129 was previously reported to carry stem rust resistance derived from *Ae. triuncialis*^8^. However, negative GISH results in this study revealed the absence of detectable *Ae. triuncialis* chromatin in Tr129 (Supplementary Fig. S1). In addition, no large linkage block normally associated with alien translocations was associated with any of the resistance genes. We concluded that the stem rust resistance in line Tr129 was not derived from *Ae*. *triuncialis*. Moreover, a genetic haplotype in the *Sr9* region similar to that of Neepawa in Tr129 did not support the reported pedigree with Marquis as the recurrent parent (Supplemental Table S1). Thus, the origin and pedigree of Tr129 is unknown.

Genetic analysis of stem rust resistance in Tr129 revealed the presence of four genes located on chromosome arms 6DS, 6AS, 4AL and 2BL. Theses genes include temporarily named *SrTr129*, *Sr8a*, *Sr7b*, and *Sr9b*, respectively. *SrTr129* conferring resistance to race A (TTKSK or Ug99, Table 1) mapped to a region known to carry resistance genes *SrCad*, *Sr42*, and *SrTmp*^13–15^. A comparison of linkage maps for *SrTr129*, *SrCad*, *Sr42*, and *SrTmp* showed collinearity and consistency in the Sr gene position on chromosome arm 6DS (Fig. 1). Since *SrTr129* conferred resistance to race C (Table 1), and Norin 40 with *Sr42* was susceptible to race C^15^, *SrTr129* cannot be *Sr42*. A line with *SrTmp* was susceptible to race TRTTF (06YEM34-1)^14^ whereas Tr129 was resistant (data not shown). *SrTr129* showed resistance to related race C (Table 1) indicating that *SrTmp* and *SrTr129* were alleles or located at different closely linked positions. Peace and Tr129 showed the same pattern of response against multiple *Pgt* races (data not presented) but since Tr129 carried four resistance genes we could not differentiate the response arrays conferred by *SrCad* and *SrTr129*. We then tested 73 random DH lines from a RL6071/Peace population used to map *SrCad*^24^ with races D, E, and F using the methods listed previously. Many lines resistant to race A (*SrCad*) were susceptible to races D, E, or F (Table 2, Supplementary table S3). It appears that Peace also has multiple *Sr* genes that need to be confirmed in future studies. Thus, *SrTr129* could be *SrCad* previously described in AC Cadillac and Peace^24^. Although we could not differentiate *SrTr129* from *SrCad* using several *Pgt* races, a comparison of SNP haplotypes for chromosome arm 6DS showed a unique haplotype for Tr129 compared to *SrCad*, *SrTmp*, and *Sr42*. However, cloning of these genes will clear the ambiguity of whether these are alleles or located at closely linked loci.

The RL6071/Tr129 DH population also segregated for a single gene that conferred resistance to race D and mapped to the chromosome arm 6AS region known to carry *Sr8* (Fig. 2). Collinearity between chromosome 6AS linkage maps in RL6071/Tr129 and LMPG-6S/Harvest DH populations^16^ support the hypothesis that resistance was conferred by an allele of *Sr8* (Fig. 2). Three resistance alleles (*Sr8a*, *Sr8b*, and *Sr8155B1*) have been reported at the *Sr8* locus^25,26^. *Sr8b* was excluded as a candidate since race D is virulent (Fetch, unpublished data). Both *Sr8a* and *Sr8155B1* conferred resistance to TRTTF^26^; the IT 2^-^ recorded in this study matched the expected response for *Sr8a* and not the IT 0; documented for *Sr8155B1*, hence the latter was excluded as a candidate.

DH lines in Subset 1 inoculated with *Pgt* race C segregated 3:1 indicating that Tr129 carries two resistance genes effective against this race (Table 1). *SrTr129* was one of these genes (Supplementary table S2), and data from Subset 2 (lines that lacked *SrTr129*) allowed mapping of another gene for race C resistance to chromosome arm 4AL (Fig. 3a). This gene was flanked by SNP markers *IWB47901* and *IWB24693* at 137.3 and 166.7 cM, respectively^17^(Supplementary table S4). The consensus map positions of *Sr7a*-linked STARP markers *Xrwgsnp10* and *Xrwgsnp11*^27^ and SNP marker *IWA1067*^28^ are close to the *Sr7* locus (Supplementary table S6). The locations of *Sr7* and *SrND643* were reported on chromosome arm 4AL^29^. Gene *SrND643* was excluded as a candidate as it was known to confer resistance to race A^29^. Since *Sr7a* is ineffective against race C it is likely that allele present in Tr129 is *Sr7b* (Table 4).

To test for the presence of *Sr9b* in Tr129, the phenotypic data for Subset 2 DH lines (lacking *SrTr129*) tested with *Pgt* races E and F was used. Although the chi squared analysis did not fit a single gene ratio for response to race E, QTL analysis indicated that only the 2BL genomic region was involved. Six *Sr* genes (*Sr9*, *Sr16*, *Sr20*, *Sr28*, *Sr47*, and *Sr883-2B* have been reported on 2BL^30,31^. As race E is virulent for *Sr16*, *Sr20*, and *Sr28* these genes were eliminated as candidates. Genes *Sr47* and *Sr883-2B* can also be eliminated as they confer resistance to race A. Thus, *Sr9b* was the only candidate allele that matched the specificity of the gene on chromosome arm 2BL in Tr129.

In conclusion, we could not identify the origin of Sr resistance in Tr129, but it was not derived from *Ae. triuncialis*. Tr129 also differed from Peace as that cultivar does not carry *Sr8a* (Hiebert, unpublished data). By using a mapping population, subsets of the population to remove confounding effects of *SrTr129*, and several *Pgt* races with differing virulence, we were able to identify and map stem rust resistance genes present in Tr129. Tr129 has at least four Sr genes: *SrTr129* located on chromosome arm 6DS, *Sr8a* on 2BL, *Sr7b* on 4AL, and *Sr9b* on 2BL. Gene *SrTr129* conferred resistance to races A, B, and C, *Sr8a* conferred resistance to races B, D, and F, *Sr7b* conferred resistance to race C, and *Sr9b* conferred resistance to races E and F. Two Sr genes in Tr129 conferring resistance to race F were reported by Ghazvini et al.^10^ (Supplementary table S2). Genes *Sr7b* and *Sr9b* are very common in Canadian wheat cultivars and provide moderate protection against individual races in the North American *Pgt* population. Gene *Sr8a* is a common source of resistance to race B in Canadian wheat cultivars^16^. Although *SrTr129* could not be differentiated from *SrCad*, Tr129 had a unique SNP haplotype in the *SrCad* genomic region and therefore could represent a new source of resistance effective against the widely virulent Ug99 lineage of *Pgt* races. Insight into the effectiveness of *SrCad* to virulent exotic races and ineffectiveness against some North American races will guide breeders on how to utilize *SrCad* in breeding for stem rust resistance.

## Material and methods

### Plant material

Tr129 is a hexaploid stem rust resistant wheat line bred by Dr. T. Aung (AAFC, retired). Wheat line RL6071 (Prelude/8*Marquis*2/3/Prelude//Prelude/8*Marquis) is a hexaploid wheat line developed by Dr. P. L. Dyck (AAFC) and was selected as a susceptible parent for genetic studies; it does not carry *Sr7b*, which is present in Marquis. We used F_2_ and doubled haploid (DH) populations developed^32^ from the cross RL6071/Tr129 to study the inheritance of stem rust resistance in Tr129 and for genetic mapping.

### Genomic in situ hybridization (GISH)

Genomic DNA of tetraploid *Aegilops triuncialis* L. (genome UUCC, 2*n* = 28) TA1752 and diploid accessions *Aegilops umbellulata* Zhuk. (genome UU, 2*n* = 14; TA1851) and *Ae*. *caudata* L. (genome CC, 2*n* = 14; TA1908) representing different subgenomes were used to prepare GISH probes. Chromosomal preparations of chromosome addition lines TA7562 (DA1U, 2*n* = 44) and 99-247-4 (DA3CtL, 2*n* = 44) were used as controls in GISH experiments. Accessions TA1752, TA1851, TA1908 and TA7562 are maintained by the Wheat Genetics Resource Center at Kansas State University. GISH was performed according to Zhang et al.^33^ with modifications described in Liu et al.^34^. Chromosome preparations were mounted and counterstained with propidium iodide (PI) in Vectashield (Vector Laboratories, Burlingham, CA, cat # H-1300). Images were captured with a Zeiss Axioplan 2 microscope using a cooled charge-coupled device camera CoolSNAP HQ2 (Photometrics, Tucson, AZ) and AxioVision 4.8 software (Zeiss). Images were processed using Adobe Photoshop software (Adobe Systems Incorporated, San Jose, CA, USA).

### Stem rust assays

All stem rust assays were performed on seedlings with fully emerged first leaves. Plants pre- and post-inoculation were grown in a greenhouse at 20 ± 2 °C with a 16 h photoperiod. Inoculation, incubation, and rating of stem rust infection types were performed as described by Hiebert et al.^24^. Briefly, *Pgt* urediniospores suspended in light mineral oil (4 mg per 0.7 ml oil) were sprayed onto seedlings. After allowing the oil to evaporate, seedlings were incubated overnight in darkness at 100% relative humidity and allowed to dry slowly under light before removal to greenhouse benches. Seedlings were rated for infection type (IT) 14 days post-inoculation following the 0–4 scale described by Stakman et al.^35^ and modified by Roelfs and Martens^36^. Six races with wide differences in virulence based on the *Pgt* letter-code nomenclature of Roelfs and Martens^36^ were used in the study to differentiate individual Sr genes. DH lines with ITs 0–2^+^ were classified resistant and ITs 3–4 were classified as susceptible. The parents (RL6071 and Tr129), F_2_ population (*n* = 85), and DH population (n =  ~ 276) were screened with *Pgt* races A (TTKSK accession SA31), E (TPMKC isolate W1373), and F (MCCFC isolate W1541). For QTL analysis, ITs were converted in to a linearized 0–9 scale^37^. Subset 1 of the DH population was randomly selected and phenotyped with *Pgt* races B (RRTTF isolate 10PAK05-1), C (QTHJF isolate W1347), and D (TMRTF isolate W1311). To better resolve the four Sr genes that were detected by multi-race testing with six *Pgt* races listed above, an additional subset (Subset 2) of DH lines lacking resistance to race A was phenotyped with race C. For the DH population or subsets of the population, three to five seedlings were rated per line for each *Pgt* race.

A multi-pathogen test was conducted with *Pgt* races D, E, and F to compare the Tr129-derived Sr gene on chromosome arm 6DS with *SrCad*. This analysis was performed on 73 randomly selected DH lines from a previously developed population from cross RL6071/Peace (BW90*3/BW553//BW90’S’/Katepwa)^24^. This population had already been phenotyped with race A. To differentiate between alleles *Sr7a* and *Sr7b*, four genotypes carrying *Sr7a* were phenotyped with the *Sr7a*-virulent *Pgt* race C along with Tr129, RL6071, and wheat line ISr7b-Ra (CI 14165)^36^ (Table 4).

### Genotyping and mapping

DNA was extracted from parent lines (RL6071 and Tr129) and F_2_ and DH progeny using a modified ammonium acetate method^38^. The F_2_ (*n* = 85) plants and parents were genotyped with the 90 K iSelect SNP array^17^. Linkage maps were constructed using MapDisto 1.8.2 (http://mapdisto.free.fr)^39^ by setting logarithm of odds (LOD) and Rmax values 3.0 and 0.3, respectively. Genetic distances were calculated with the Kosambi mapping function^40^. After initial mapping of the Sr gene that conferred resistance to *Pgt* race A to chromosome arm 6DS in the F_2_ population, further analyses were undertaken using the DH population (*n* = 276). *SrCad* region-specific kompetitive allele specific PCR (KASP) markers on chromosome arm 6DS for genotyping were selected from Kassa et al.^13^ and analysis of those markers followed the same publication^13^. In addition, two simple sequence repeat (SSR) markers (*cfd49* and *gpw5182*) and common bunt resistance gene (*Bt*-10) PCR marker *FSD_RSA*^41^ were also included in the chromosome 6DS linkage map. Genotyping of the DH population with marker *FSD_RSA* followed procedures described by Hiebert et al.^24^. SSR genotyping was done following described procedures^42,43^. PCR products were analysed by using an ABI 3100 genetic analyzer (Applied Biosystems, Streetsville, ON, Canada). Wheat lines Triumph 64, Tr129, Peace, and Norin 40 were haplotyped with 14 KASP markers (*kwm112, kwm191, kwm196, kwm197, kwm244, kwm871, kwm873, kwm918, kwm929, kwm987, kwm994, kwm997, kwm999,* and *kwm1000*) described by Kassa et al.^13^ to characterize the region spanning *SrCad* in chromosome arm 6DS.

To map additional Sr genes present in the Tr129 line, further genetic analysis was done by using two different subsets of the DH population as explained earlier. Subset 1 contained randomly selected DH lines, and Subset 2 consisted of DH lines lacking resistance for race A. DH lines from Subset 1 were used to map two additional Sr genes present in Tr129. Subset 2 was used to map a race E-specific resistance gene from Tr129. Subset 2 was also phenotyped with the race C to support the genotypic analysis conducted on Subset 1.

For mapping the Sr gene effective against *Pgt* races B, D, and F, we first considered chromosome arm 6AS based on the initial analysis done by Ghazvini et al.^11^ with race F. To develop a chromosome 6AS linkage map for the Tr129 DH population, nine KASP markers (*kwh53*, *kwh54*, *kwh58*, *kwh62*, *kwh63*, *kwh223*, *kwh225*, *kwh227*, and *kwh233*) were selected from Hiebert et al.^16^ (Supplementary table S7). The 6AS linkage map was developed with these nine KASP markers, and phenotypic data from races B, D, and F was used to map an additional Sr gene derived from Tr129.

90 K iSelect genotyping was performed on Subset 2 DH lines to determine the chromosomal locations of two additional Sr genes in Tr129. QTL analysis was carried out using QGENE 4.4.0 (https://www.qgene.org/)^44^ and single-trait multiple interval mapping (MIM) was used to detect genomic regions associated with specific races^45^. After initial QTL detection, the phenotypes for races C and E were mapped as Mendelian traits. Mapchart 2.32 (https://www.wur.nl/en/show/mapchart.htm)^46^ was used to develop the linkage map figures. After locating the Sr gene on chromosome arm 4AL, polymorphic iSelect SNPs were converted to KASP markers (Supplementary table S7). An additional KASP marker (*kwh703*) was developed from the *Sr7* region-associated SNP-based semi-thermal asymmetric reverse PCR (STARP) marker *Xrwgsnp11* from Saini et al.^27^ (Supplementary table S7). An Sr gene effective against races E and F was identified on chromosome arm 2BL, and 21 KASP markers were developed from polymorphic iSelect SNPs identified on chromosome 2B. DNA marker genotyping and linkage mapping for all Sr genes were performed according to the procedure described above. An additional genotypic analysis was done on the parental lines Tr129 and RL6071, as well as Neepawa and Marquis, with eight SSR markers (Supplementary table S1) specific for the *Sr9* region^23,43,47^.

### Research involving plants

All field experiments were in compliance with Institutional, National and International guideline policies.

## Supplementary Information

Supplementary Information.

## Data Availability

SNP marker data and raw infection type data are available upon request.

## References

[1] Pretorius, Z. A., Singh, R. P., Wagoire, W.W. & Payne, T. S. Detection of virulence to wheat stem rust resistance gene *Sr31* in *Puccinia graminis* f. sp. *tritici* in Uganda. *Plant Dis.* **84**, 203 (2000). 10.1094/PDIS.2000.84.2.203B 30841334

[2] Jin Y (2005). Races of *Puccinia graminis* identified in the United States during 2003. Plant Dis..

[3] Fetch T (2012). Virulence of Ug99 (race TTKSK) and race TRTTF on Canadian wheat cultivars. Can. J. Plant Sci..

[4] Olivera, P. D. *et al.* Races of *Puccinia graminis* f. sp. *tritici* with combined virulence to *Sr13* and *Sr9e* in a field stem rust screening nursery in Ethiopia. *Plant Dis*. **96**, 623–628 (2012). 10.1094/PDIS-09-11-0793 30727519

[5] Olivera, P. *et al.* Phenotypic and genotypic characterization of race TKTTF of *Puccinia graminis* f. sp. *tritici* that caused a wheat stem rust epidemic in southern Ethiopia in 2013–14. *Phytopathology* **105**, 917–928 (2015). 10.1094/PHYTO-11-14-0302-FI 25775107

[6] Olivera Firpo, P. D. *et al.* Characterization of *Puccinia graminis* f. sp. *tritici* isolates derived from an unusual wheat stem rust outbreak in Germany in 2013. *Plant Pathol*. **66**, 1258–1266 (2017).

[7] Patpour, M., Hovmøller, M. S. & Hodson, D. First report of virulence to *Sr25* in race TKTTF of *Puccinia graminis* f. sp. *tritici* causing stem rust on wheat. *Plant Dis*. *Notes*10.1094/PDIS-11-16-1666-PDN (2017).

[8] Aung T, Kerber ER (1994). Incorporation of leaf rust resistance from wild tetraploid into cultivated hexaploid wheat. Ann. Wheat Newslet..

[9] Fetch, T. & Zegeye, T. Inheritance of resistance to Ug99 in wheat line Tr129 with an introgression of *Aegilops triuncialis* chromatin. Page 33. Proceedings of 12th International Cereal Rusts and Powdery Mildews Conference. October 13–16, 2009 Antalya, Turkey.

[10] Ghazvini H, Hiebert CW, Zegeye T, Fetch T (2012). Inheritance of stem rust resistance derived from *Aegilops triuncialis* in wheat line Tr129. Can. J. Plant Sci..

[11] Ghazvini, H., Hiebert, C. W., Thomas, J., Zegeye, T. & Fetch, T. Linkage maps of two new stem rust resistance genes on chromosomes 2B and 6A of wheat line Tr129. Page 602–603. Abstracts of technical papers presented at the 1st Canadian wheat symposium. November 30–December 2, 2011 Winnipeg, Manitoba, Canada.

[12] Kolmer JA, Dyck PL, Roelfs AP (1991). An appraisal of stem and leaf rust resistance in North American hard red spring wheats and the probability of multiple mutations in populations of cereal rust fungi. Phytopathology.

[13] Kassa MT (2016). Genetic mapping of *SrCad* and SNP marker development for marker-assisted selection of Ug99 stem rust resistance in wheat. Theor. Appl. Genet..

[14] Hiebert CW (2016). Genetics and mapping of seedling resistance to Ug99 stem rust in winter wheat cultivar Triumph 64 and differentiation of *SrTmp*, *SrCad*, and *Sr42*. Theor. Appl. Genet..

[15] Ghazvini H (2012). Inheritance of resistance to Ug99 stem rust in wheat cultivar Norin 40 and genetic mapping of *Sr42*. Theor. Appl. Genet..

[16] Hiebert, C. W., Rouse, M. N., Nirmala, J. & Fetch, T. Genetic mapping of stem rust resistance to *Puccinia graminis* f. sp. *tritici* race TRTTF in the Canadian wheat cultivar Harvest. *Phytopathology* **107**, 192–197 (2017). 10.1094/PHYTO-05-16-0186-R 27705664

[17] Wang S (2014). Characterization of polyploid wheat genomic diversity using a high-density 90 000 single nucleotide polymorphism array. Plant Biotechnol. J..

[18] Green GJ, Knott DR, Watson IA, Pugsley AT (1960). Seedling reactions to stem rust of lines of Marquis wheat with substituted genes for rust resistance. Can. J. Plant Sci..

[19] Hiebert CW, Fetch TG, Zegeye T (2010). Genetics and mapping of stem rust resistance to Ug99 in the wheat cultivar Webster. Theor. Appl. Genet..

[20] Knott, D. R. The inheritance of stem rust resistance in wheat. In *Proc. 2*^*nd*^* International Wheat Genetics Symposium* 156–166 (1963).

[21] McIntosh, R. A. & Luig, N. H. Recombination between genes for reaction to *P*. *graminis* at or near the *Sr9* locus. In *Proc. 4th International Wheat Genetics Symposium*. 425–432 (1973).

[22] Loegering WQ (1975). An allele for low reaction to *Puccinia graminis tritici* in Chinese Spring wheat. Phytopathology.

[23] Rouse MN (2014). Characterization of *Sr9h*, a wheat stem rust resistance allele effective to Ug99. Theor. Appl. Genet..

[24] Hiebert CW (2011). Genetics and mapping of seedling resistance to Ug99 stem rust in Canadian wheat cultivars ‘Peace’ and ‘AC Cadillac’. Theor. Appl. Genet..

[25] McIntosh RA, Welling CR, Park RF (1995). Wheat rusts: An atlas of resistance genes.

[26] Nirmala, J. *et al*. Discovery of a novel stem rust resistance allele in durum wheat that exhibits differential reactions to Ug99 isolates. *G3 (Bethesda)* **7**, 3481–3490 (2017). 10.1534/g3.117.300209 PMC5633396 28855282

[27] Saini J (2018). Identification, mapping, and marker development of stem rust resistance genes in durum wheat ‘Lebsock’. Mol. Breed..

[28] Turner MK, Jin Y, Rouse MN, Anderson JA (2016). Stem rust resistance in ‘Jagger’ winter wheat. Crop Sci..

[29] Basnet BR (2015). Molecular mapping and validation of *SrND643*: A new wheat gene for resistance to the stem rust pathogen Ug99 race group. Phytopathology.

[30] McIntosh, R.A. *et al*. Catalogue of gene symbols for wheat, 2013 edition. Online at: https://shigen.nig.ac.jp/wheat/komugi/genes/macgene/2013/GeneSymbol.pdf .

[31] Sharma JS (2019). Mapping and characterization of two stem rust resistance genes derived from cultivated emmer wheat accession PI 193883. Theor. Appl. Genet..

[32] Thomas J, Chen Q, Howes N (1997). Chromosome doubling of haploids of common wheat with caffeine. Genome.

[33] Zhang P, Friebe B, Lukaszewski AJ, Gill BS (2001). The centromere structure in Robertsonian wheat-rye translocation chromosomes indicates that centric breakage-fusion can occur at different positions within the primary constriction. Chromosoma.

[34] Liu W, Seifers DL, Qi LL, Friebe B, Gill BS (2011). A compensating wheat - *Thinopyrum intermedium* Robertsonian translocation conferring resistance to *wheat streak mosaic virus* and *Triticum* mosaic virus. Crop Sci..

[35] Stakman, E. C., Stewart, D. M. & Loegering, W. Q. Identification of physiologic races of *Puccinia graminis* var*. tritici.* USDA ARS E-617. U.S. Gov. Print. Off., Washington, DC (1962).

[36] Roelfs, A. P. & Martens, J. W. An international system of nomenclature for *Puccinia graminis* f. sp. *tritici*. *Phytopathology* **78**, 526–533 (1988).

[37] Zhang, D., Bowden, R. L., Yu, J., Carver, B. F. & Bai, G. Association analysis of stem rust resistance in U.S. winter wheat. *PLoS One* **9**:e103747. 10.1371/journal.pone.0103747 (2014). 10.1371/journal.pone.0103747 PMC4114971 25072699

[38] Pallotta, M. A. *et al.* Marker assisted wheat breeding in the southern region of Australia. In *Proc. 10th International Wheat Genetics Symposium*. 789–791 (2003).

[39] Lorieux M (2012). MapDisto: fast and efficient computation of genetic linkage maps. Mol. Breed..

[40] Kosambi DD (1943). The estimation of map distances from recombination values. Ann. Eugen..

[41] Laroche A (2000). Development of a PCR marker for rapid identification of the Bt-10 gene for common bunt resistance in wheat. Genome.

[42] Somers, D. J., Isaac, P. & Edwards, K. A high density microsatellite consensus map for bread wheat (*Triticum aestivum* L.). *Theor*. *Appl*. *Genet*. **109**, 1105–1114 (2004). 10.1007/s00122-004-1740-7 15490101

[43] Sourdille, P. *et al.* Microsatellite-based deletion bin system for the establishment of genetic-physical map relationships in wheat (*Triticum aestivum* L.). *Funct. Integr. Genomics* **4**, 12–25 (2004). 10.1007/s10142-004-0106-1 15004738

[44] Joehanes, R. & Nelson, J. C. QGene 4.0, an extensible Java QTL-analysis platform. *Bioinformatics* **24**, 2788–2789 (2008). 10.1093/bioinformatics/btn523 18940826

[45] Kao CH, Zeng ZB, Teasdale RD (1999). Multiple interval mapping for quantitative trait loci. Genetics.

[46] Voorrips RE (2002). MapChart: Software for the graphical presentation of linkage maps and QTLs. J. Hered..

[47] Sourdille, P. *et al.* Wheat Génoplante SSR mapping data release: a new set of markers and comprehensive genetic and physical mapping data http://wheat.pw.usda.gov/ggpages/SSRclub/GeneticPhysical/. Accessed 25 June 2021 (2010).

