# Supplementary material for: Origin and genetic analysis of stem rust resistance in wheat line Tr129
Source: Sci Rep. 2022 Mar 17;12:4585. doi: 10.1038/s41598-022-08681-4 (PMC8931155; doi:10.1038/s41598-022-08681-4)
Supplement: Supplementary file 1 — Supplementary Information. [file 41598_2022_8681_MOESM1_ESM.pdf]

Supplementary figure legends:

Supplementary figure 1: Panels A-C: GISH on control lines (A) 99-247-4 (DA3CtL, 2n = 44) and (B and C)TA7562 (DA1U, 2n = 44) with probes produced from genomic DNA of *Ae. caudata* (pAc), *Ae. triuncialis* (pAtr) and *Ae. umbellulata* (pAu), respectively, that detected alien chromatin. Panels D-F: GISH on Tr129 with probes D - pAc, E - pAtr and F - pAu failed to detect presence of alien chromatin. Wheat chromatin counterstained with propidium iodide is shown in red, alien chromatin is green; E, F - GAA repeats are shown in white. Scale bars = 10µm.

Supplementary figure 2: Infection types (ITs) produced on seedlings of parents RL6071 and Tr129 and doubled haploid lines at 14 days post-inoculation with (a) *Pgt* race F (MCCFC) and (b) race E (TPMKC).

Supplementary figure 3: Infection types (ITs) produced on seedlings of parents RL6071 and Tr129 and wheat lines carrying either *Sr7a*/*7b* alleles at 14 days post-inoculation with *Pgt* race C (QTHJF). Lines 1-4 were DK3, Green Na 101X Mq*6, EgNa101/*6 Mq 1-4-3, and Mq*6/ K117A, respectively.

Supplementary tables legends

Supplementary table 1: Comparison of SSR size in Tr129, Neepawa, RL6071, and Marquis for the region carrying *Sr9*. The total number of alleles for stocks carrying *Sr9a*, *Sr9b*, *Sr9d*, *Sr9e*, *Sr9f*, *Sr9g* and *Sr9h* was also tabulated for each SSR marker

Supplementary table 2: Stem rust reaction of 95 RL6071×Tr129 doubled haploid lines for *Pgt* races D (TMRTF), A (TTKSK), B (RRTTF), C (QTHJF), F (MCCFC), and E (TPMKC)

Supplementary table 3: Stem rust reactions of RL6071 × Peace doubled haploid population for *Pgt* races D (TMRTF), F (MCCFC), E (TPMKC), and A (TTKSK)

Supplementary table 4: Reference map locations (cM) of SNP markers flanking *SrTr129-4A*

Supplementary table 5: Chromosome 2B linkage map developed for the RL6071×Tr129 doubled haploid Subset 2 population lacking resistance to race A (TTKSK)

Supplementary table 6: Comparison of consensus map locations of SNP markers located in the *Sr7*-region from three different mapping studies

Supplementary table 7: Kompetitive allele specific (KASP) primer name, SNP name, chromosome location, and primer sequence information for markers in the *Sr7* (2BL), *Sr8a* (6A), and *Sr9* (2BL) chromosome regions

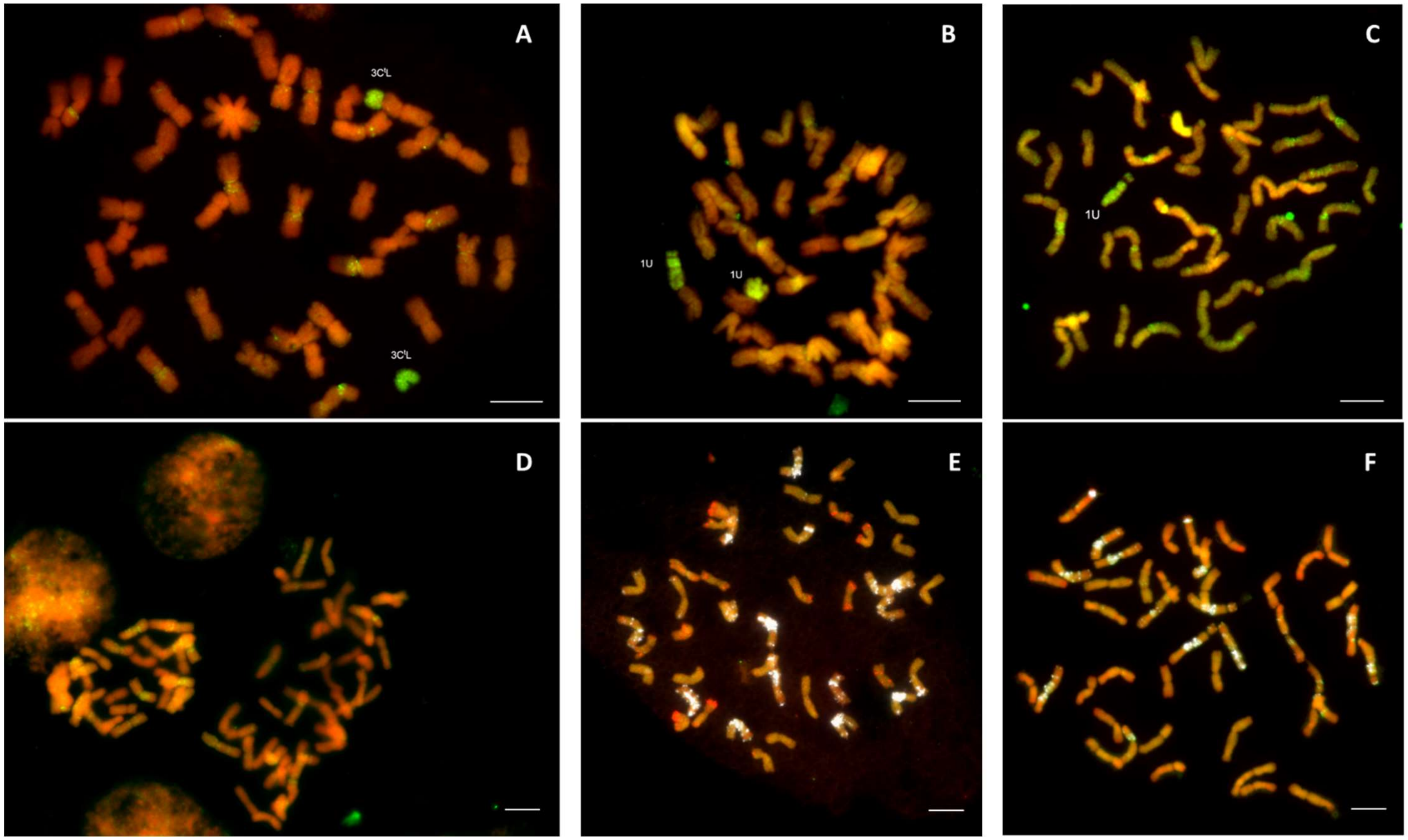

Supplementary figure 1: Panels A-C: GISH on control lines (A) 99-247-4 (DA3CtL, 2n = 44) and (B and C)TA7562 (DA1U, 2n = 44) with probes produced from genomic DNA of *Ae. caudata* (pAc), *Ae. triuncialis* (pAtr) and *Ae. umbellulata* (pAu), respectively, that detected alien chromatin. Panels D-F: GISH on Tr129 with probes D - pAc, E - pAtr and F - pAu failed to detect presence of alien chromatin. Wheat chromatin counterstained with propidium iodide is shown in red, alien chromatin is green; E, F - GAA repeats are shown in white. Scale bars = 10µm.

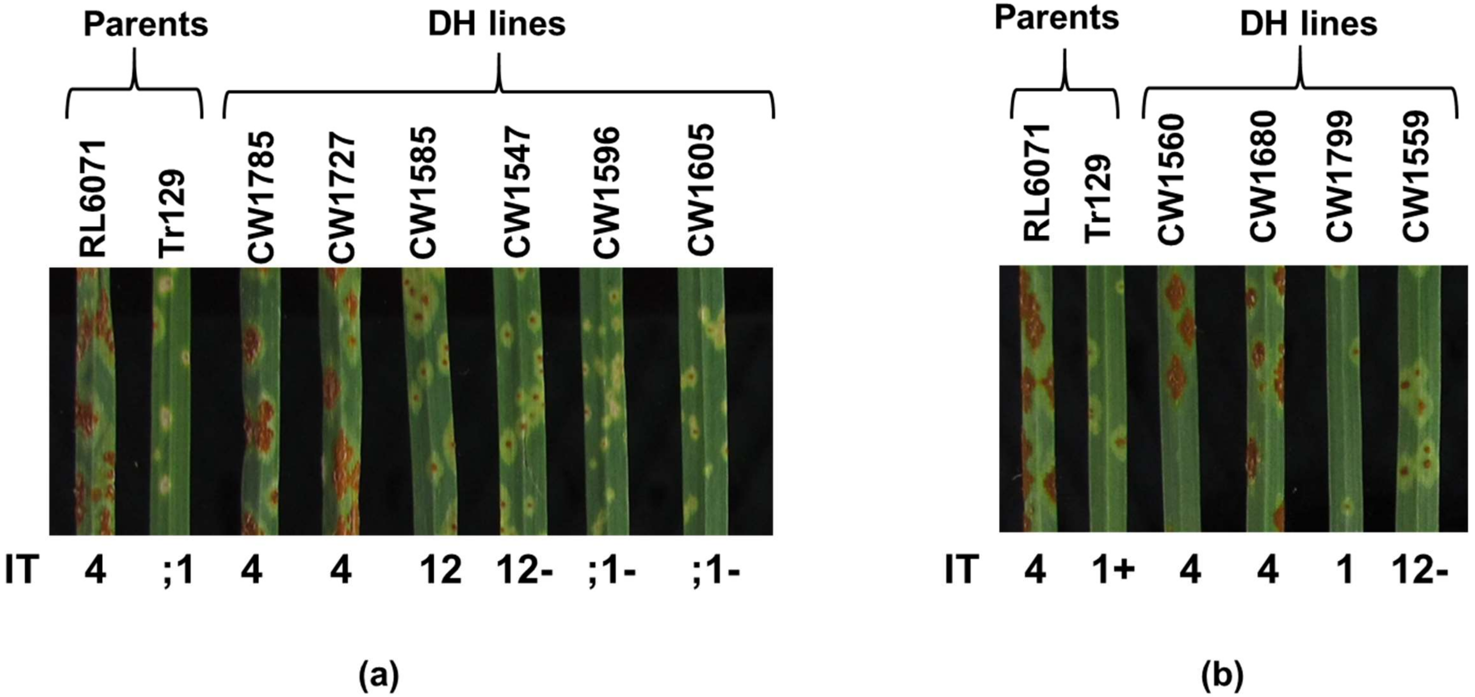

Supplementary figure 2: Infection types (ITs) produced on seedlings of parents RL6071 and Tr129 and doubled haploid (DH) lines at 14 days post-inoculation with (a) *Pgt* race F (MCCFC) and (b) race E (TPMKC).

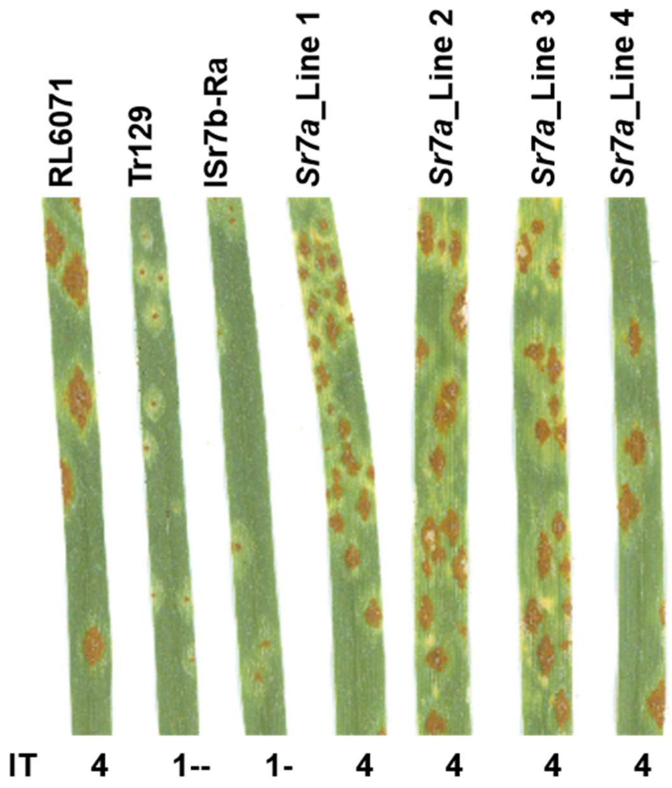

Supplementary figure 3: Infection types (ITs) produced on seedlings of parents RL6071 and Tr129 and wheat lines carrying either *Sr7a*/*7b* alleles at 14 days post-inoculation with *Pgt* race C (QTHJF). Lines 1-4 were DK3, Green Na 101X Mq*6, EgNa101/*6 Mq 1-4-3, and Mq*6/ K117A, respectively.

Supplementary table 1: Comparison of SSR size in Tr129, Neepawa, RL6071, and Marquis for the region carrying *Sr9*. The total number of alleles for stocks carrying *Sr9a*, *Sr9b*, *Sr9d*, *Sr9e*, *Sr9f*, *Sr9g* and *Sr9h* was also tabulated for each SSR marker

SSR	Allele size (bp)				Number of alleles in *Sr9* stocks
	Tr129	Neepawa	RL6071	Marquis	
gpw7506	342	342	352	352	4
gpw4396	160	160	164	164	4
cfd73	302	302	300	290	6
wmc175	268	268	268	268	2
gwm47	160	160	164	164	4
wmc332	192	192	null	233	6
gpw4112	264	264	224	266	7
gpw4103	298	298	294	296	5

Supplementary table 2: Stem rust reaction of 95 RL6071×Tr129 doubled haploid lines for *Pgt* races D (TMRTF), A (TTKSK), B (RRTTF), C (QTHJF), F (MCCFC), and E (TPMKC)

DH Line no#	TMRTF (D)	TTKSK (A)	RRTTF (B)	QTHJF (C)	MCCFC (F)	TPMKC (E)
CW1535	S	R	R	R	S	S
CW1536	S	S	S	S	S	S
CW1537	S	S	S	S	S	S
CW1538	R	S	R	R	R	S
CW1540	R	S	R	S	R	S
CW1542	R	R	R	R	R	R
CW1548	S	R	R	R	S	S
CW1549	R	S	R	R	R	S
CW1550	S	S	S	S	S	S
CW1551	S	R	R	R	R	R
CW1557	R	R	R	R	R	R
CW1558	S	R	R	R	S	S
CW1559	R	R	R	R	R	R
CW1560	R	S	R	S	R	S
CW1565	R	R	R	R	R	R
CW1569	R	R	R	R	R	R
CW1571	S	R	R	R	R	R
CW1572	S	S	S	S	R	R
CW1575	S	S	C	R	S	S
CW1577	R	S	R	R	R	R
CW1579	R	R	R	R	R	R
CW1580	R	R	R	R	R	S
CW1581	S	S	S	S	R	R
CW1585	S	R	R	R	R	R
CW1586	S	S	S	S	S	S
CW1587	R	R	R	R	R	S
CW1588	S	S	S	R	S	S
CW1589	S	S	C	R	S	S
CW1591	R	S	R	S	R	S
CW1595	R	R	R	R	R	S
CW1596	R	R	R	R	R	R
CW1597	R	R	R	R	R	S
CW1598	S	R	R	R	R	R

CW1600	R	R	R	R	R	S
CW1601	R	R	R	R	R	R
CW1602	R	R	R	R	R	R
CW1608	S	R	R	R	R	R
CW1611	S	S	S	R	R	R
CW1612	S	R	R	R	S	S
CW1613	R	R	R	R	R	S
CW1614	S	R	R	R	R	R
CW1617	S	R	R	R	R	R
CW1618	S	R	R	R	R	R
CW1620	R	R	R	R	R	S
CW1621	S	R	R	R	C	S
CW1622	R	S	R	S	R	S
CW1625	R	R	R	R	R	S
CW1627	S	S	C	R	R	R
CW1628	S	S	S	R	S	S
CW1629	R	R	R	R	R	R
CW1632	R	R	R	R	R	S
CW1633	R	S	R	S	R	R
CW1634	R	S	R	R	R	R
CW1635	S	S	S	R	S	S
CW1636	S	S	S	S	S	S
CW1637	S	S	S	R	S	S
CW1639	R	R	R	R	R	S
CW1640	S	R	R	R	R	R
CW1642	S	S	S	R	S	S
CW1644	R	R	R	R	R	R
CW1645	R	R	R	R	R	S
CW1647	R	S	R	S	R	R
CW1648	R	R	R	R	R	S
CW1650	R	S	R	S	R	R
CW1657	S	S	S	R	S	S
CW1658	R	R	R	R	R	R
CW1660	R	R	R	R	R	R
CW1661	S	S	S	R	S	S
CW1663	R	S	R	S	R	S
CW1668	R	S	R	R	R	S
CW1670	R	S	R	R	R	S

CW1673	R	R	R	R	R	R
CW1675	S	R	R	R	S	S
CW1678	R	S	R	S	R	S
CW1681	S	S	C	S	S	S
CW1682	S	R	R	R	R	R
CW1686	R	R	R	R	R	R
CW1687	R	S	R	R	R	S
CW1688	S	R	R	R	R	R
CW1691	R	S	R	S	R	S
CW1693	R	R	R	R	R	R
CW1695	R	S	R	S	R	S
CW1698	S	S	C	S	S	S
CW1699	R	S	R	R	R	S
CW1700	R	S	R	S	R	R
CW1701	R	R	R	R	R	R
CW1702	R	S	R	S	R	S
CW1703	R	R	R	R	R	R
CW1704	S	S	C	R	S	S
CW1705	S	S	S	S	S	S
CW1710	S	S	S	S	S	S
CW1712	S	S	S	R	R	R
CW1716	S	S	S	S	S	R
CW1717	S	S	S	R	S	S
CW1719	S	S	S	R	S	S
**Total R**	51	47	68	70	67	41
**Total S**	44	48	21	25	27	54
**Chi square (χ^2)**	0.52	0.01	0.09	0.08	0.69	1.779
**Probability *(p)***	0.47	0.92	0.76	0.77	0.4	0.18
**No. of gene**	1	1	2	2	2	

R = Resistant, S = Suscptible, C = Missing data

Supplementary table 3: Stem rust reactions of RL6071 × Peace doubled haploid population for *Pgt* races D (TMRTF), F (MCCFC), E (TPMKC), and A (TTKSK)

DH line no.	Source	TMRTF	MCCFC	TPMKC	TTKSK
CZ1041	06B01*A0002	S	R	R	S
CZ1042	06B01*A0015	S	R	R	S
CZ1043	06B01*A0016	R	S	S	S
CZ1045	06B01*A0021	R	S	S	R
CZ1046	06B01*A0034	S	R	R	R
CZ1047	06B01*A0037	R	R	R	S
CZ1048	06B01*A0038	R	S	S	S
CZ1050	06B01*A0046	R	S	S	R
CZ1051	06B01*A0054	R	R	R	R
CZ1052	06B01*A0076	R	S	S	S
CZ1053	06B01*A0080	S	S	S	R
CZ1054	06B01*A0092	S	S	S	S
CZ1055	06B01*A0098	R	S	S	S
CZ1056	06B01*A0190	R	R	R	R
CZ1058	06B01*A0197	S	S	S	S
CZ1060	06B01*A0205	R	R	R	R
CZ1062	06B01*A0266	S	S	S	R
CZ1063	06B01*A0282	S	S	S	R
CZ1064	06B01*A0313	R	R	R	S
CZ1065	06B01*A0318	S	R	R	S
CZ1070	06B01*B0104	R	R	R	R
CZ1072	06B01*B0209	R	S	S	R
CZ1075	06B01*B0330	R	R	R	R
CZ1076	06B01*B0331	R	R	R	S
CZ1080	06B01*B0441	S	S	S	S
CZ1083	06B01*B0618	S	R	R	S
CZ1085	06B01*B0738	R	S	S	R
CZ1086	06B01*B0779	R	S	S	S
CZ1090	06B01*C0072	C	R	R	S
CZ1091	06B01*C0112	R	R	R	S

CZ1092	06B01*C0237	S	S	S	R
CZ1093	06B01*C0238	R	S	S	S
CZ1096	06B01*C0366	S	S	S	S
CZ1097	06B01*C0459	S	S	S	S
CZ1098	06B01*C0465	S	S	S	R
CZ1099	06B01*C0467	S	R	R	S
CZ1100	06B01*C0474	S	S	S	S
CZ1101	06B01*C0477	R	S	S	S
CZ1102	06B01*C0487	R	R	R	S
CZ1103	06B01*C0489	S	R	R	R
CZ1104	06B01*C0642	R	S	S	R
CZ1106	06B01*C0792	S	R	R	R
CZ1107	06B01*D0130	R	R	R	R
CZ1108	06B01*D0136	R	S	S	S
CZ1109	06B01*D0137	R	R	R	R
CZ1111	06B01*D0256	S	R	R	R
CZ1112	06B01*D0260	S	S	S	S
CZ1114	06B01*D0492	S	S	S	R
CZ1116	06B01*D0505	S	R	R	R
CZ1119	06B01*D0653	C	S	S	R
CZ1123	06B01*D0748	R	R	R	S
CZ1124	06B01*D0751	S	R	R	R
CZ1127	06B01*E0184	S	R	R	S
CZ1128	06B01*E0185	S	R	R	R
CZ1129	06B01*E0187	S	R	R	S
CZ1130	06B01*E0188	S	S	S	R
CZ1132	06B01*E0300	S	R	R	R
CZ1133	06B01*E0303	S	S	S	R
CZ1134	06B01*E0383	R	S	S	R
CZ1135	06B01*E0388	R	S	S	S
CZ1136	06B01*E0391	S	S	S	S
CZ1137	06B01*E0392	R	R	R	S
CZ1138	06B01*E0548	S	R	R	R
CZ1139	06B01*E0554	S	R	R	S

CZ1140	06B01*E0561	R	R	R	R
CZ1141	06B01*E0574	R	C	C	S
CZ1142	06B01*E0583	R	R	R	R
CZ1143	06B01*E0587	R	S	S	R
CZ1146	06B01*E0765	R	R	R	S
CZ1147	06B01*E0766	S	R	R	S
CZ1148	06B01*E0774	R	R	R	S
CZ1149	06B01*E0804	R	R	R	S
CZ1150	06B01*A0007	R	R	R	S

R = Resistant, S = Suscptible, C = Missing data

Supplementary table 4: Reference map locations (cM) of SNP markers flanking *SrTr129-4A*

Index no.	SNP id	SNP Name	Chr.	cM
47901.00	IWB47901	Kukri_c81845_114	4A	52.1
11801.00	IWB11801	BS00093289_51	4A	52.1
8193.00	IWB8193	BS00039811_51	4A	52.1
71640.00	IWB71640	Tdurum_contig45738_670	4A	52.1
23841.00	IWB23841	Excalibur_c22708_625	4A	63.1
19553.00	IWB19553	Ex_c101560_2288	4A	63.1
24693.00	IWB24693.1	Excalibur_c2827_580	4A	63.1
34249.00	IWB34249	GENE-4923_84	4A	63.1
26814.00	IWB26814	Excalibur_c46812_161	4A	63.1
52406.00	IWB52406	Ra_c6672_1679	4A	63.1
12229.00	IWB12229	BS00104640_51	4A	63.1
76446.00	IWA1674	wsnp_Ex_c12725_20212708	4A	63.1
60692.00	IWB60692	RAC875_c82470_174	4A	63.1
71808.00	IWB71808	Tdurum_contig47476_441	4A	63.1
71979.00	IWB71979	Tdurum_contig49559_2160	4A	63.1
70303.00	IWB70303	Tdurum_contig31852_251	4A	63.1
80365.00	IWA6878	wsnp_Ku_c3056_5734773	4A	63.1
80004.00	IWA6392	wsnp_Ku_c10434_17255840	4A	63.1
8301.00	IWB8301	BS00042071_51	4A	63.1
76581.00	IWA1836	wsnp_Ex_c13953_21832185	4A	63.1
71693.00	IWB71693	Tdurum_contig46583_1275	4A	63.1
31512.00	IWB31512	Excalibur_s108810_150	4A	63.1
45728.00	IWB45728	Kukri_c4709_53	4A	63.1
71544.00	IWB71544	Tdurum_contig44771_984	4A	63.1
72931.00	IWB72931	Tdurum_contig63050_264	4A	63.1
77780.00	IWA3422	wsnp_Ex_c33012_41567026	4A	63.1
67739.00	IWB67739	Tdurum_contig12802_331	4A	63.1
73720.00	IWB73720	Tdurum_contig8712_129	4A	63.1
81157.00	IWA7939	wsnp_Ra_c4400_7986499	4A	63.1
70192.00	IWB70192	Tdurum_contig31218_208	4A	63.1
70193.00	IWB70193	Tdurum_contig31218_279	4A	63.1
29925.00	IWB29925	Excalibur_rep_c103261_161	4A	63.1
53285.00	IWB53285	RAC875_c11237_357	4A	63.1
6928.00	IWB6928	BS00022146_51	4A	63.1
73933.00	IWB73933	Tdurum_contig9530_69	4A	63.1
64428.00	IWB64428	RFL_Contig3621_947	4A	63.1
64422.00	IWB64422	RFL_Contig3621_1157	4A	63.1
70194.00	IWB70194	Tdurum_contig31218_344	4A	63.1

275.00	IWB275	BobWhite_c11918_807	4A	63.1
44828.00	IWB44828	Kukri_c3866_1768	4A	63.1
4517.00	IWB4517	BobWhite_c877_1058	4A	63.1
48829.00	IWB48829	Kukri_rep_c102971_140	4A	63.1
6097.00	IWB6097	BS00009680_51	4A	63.1
72930.00	IWB72930	Tdurum_contig63050_181	4A	63.1
76237.00	IWA1410	wsnp_Ex_c10955_17794520	4A	63.1
79306.00	IWA5353	wsnp_Ex_rep_c67099_65575038	4A	63.1
67745.00	IWB67745.1	Tdurum_contig12832_116	4A	63.1
78768.00	IWA4689.1	wsnp_Ex_c7489_12810046	4A	63.1
63978.00	IWB63978.1	RFL_Contig2531_969	4A	63.1
34478.00	IWB34478	IAAV1943	4A	63.1
1867.00	IWB1867	BobWhite_c25015_1218	4A	63.1
77591.00	IWA3192	wsnp_Ex_c2886_5325103	4A	63.1
66655.00	IWB66655	Tdurum_contig10654_704	4A	63.1
73467.00	IWB73467	Tdurum_contig80287_71	4A	63.1
12146.00	IWB12146	BS00100763_51	4A	63.1
71695.00	IWB71695	Tdurum_contig46583_1457	4A	63.1
69385.00	IWB69385	Tdurum_contig27944_266	4A	63.1
5669.00	IWB5669	BobWhite_s64194_60	4A	63.1
61749.00	IWB61749	RAC875_rep_c107984_318	4A	63.1
74069.00	IWB74069	Tdurum_contig99556_220	4A	63.1
74014.00	IWB74014	Tdurum_contig97806_76	4A	63.1
80735.00	IWA7364	wsnp_Ku_c9746_16265584	4A	63.1
80736.00	IWA7365	wsnp_Ku_c9746_16265756	4A	63.1
25418.00	IWB25418.1	Excalibur_c34115_727	4A	63.1
27971.00	IWB27971	Excalibur_c6050_323	4A	63.1
63975.00	IWB63975	RFL_Contig2531_1872	4A	63.1
48738.00	IWB48738	Kukri_rep_c102191_135	4A	63.1
19550.00	IWB19550	Ex_c101546_376	4A	63.1
57180.00	IWB57180	RAC875_c36363_222	4A	63.1
19549.00	IWB19549	Ex_c101546_128	4A	63.1
76314.00	IWA1505	wsnp_Ex_c11474_18507872	4A	63.1
74281.00	IWB74281	tplb0026e22_471	4A	63.1
15031.00	IWB15031	CAP8_rep_c4703_313	4A	63.1
39026.00	IWB39026	Ku_c27642_444	4A	63.1
42226.00	IWB42226	Kukri_c19883_629	4A	63.1
22848.00	IWB22848	Excalibur_c16775_1734	4A	63.1
36777.00	IWB36777	Jagger_c8310_70	4A	63.1
42225.00	IWB42225	Kukri_c19883_365	4A	63.1
8552.00	IWB8552	BS00049695_51	4A	63.1
5608.00	IWB5608	BobWhite_rep_c66361_594	4A	63.1
71810.00	IWB71810	Tdurum_contig47476_528	4A	63.1
9157.00	IWB9157	BS00063943_51	4A	63.1
1158.00	IWB1158	BobWhite_c18267_473	4A	63.1

12593.00	IWB12593	BS00110758_51	4A	63.1
80101.00	IWA6518	wsnp_Ku_c14220_22456455	4A	63.1
22898.00	IWB22898	Excalibur_c17006_73	4A	63.1
50393.00	IWB50393	Kukri_rep_c80771_409	4A	63.1
59346.00	IWB59346	RAC875_c58134_239	4A	63.1
46888.00	IWB46888	Kukri_c6091_327	4A	63.1
71319.00	IWB71319	Tdurum_contig42638_531	4A	63.1
23333.00	IWB23333	Excalibur_c1952_994	4A	63.1
8062.00	IWB8062	BS00036649_51	4A	63.1
33887.00	IWB33887	GENE-4290_907	4A	63.1
34594.00	IWB34594	IAAV2678	4A	63.1
43885.00	IWB43885	Kukri_c30693_573	4A	63.1
47019.00	IWB47019	Kukri_c6282_1706	4A	63.1
24532.00	IWB24532	Excalibur_c27082_65	4A	63.1
1389.00	IWB1389	BobWhite_c20382_117	4A	63.1
57392.00	IWB57392	RAC875_c38350_106	4A	63.1
58574.00	IWB58574	RAC875_c4965_1523	4A	63.1
76580.00	IWA1835	wsnp_Ex_c13953_21831752	4A	63.1
22948.00	IWB22948	Excalibur_c17294_228	4A	63.1
60880.00	IWB60880	RAC875_c8714_1139	4A	63.1
23633.00	IWB23633	Excalibur_c21395_291	4A	63.1
29736.00	IWB29736	Excalibur_rep_c101560_1124	4A	63.1
11060.00	IWB11060	BS00079092_51	4A	63.1
11988.00	IWB11988	BS00097194_51	4A	63.1
30794.00	IWB30794	Excalibur_rep_c66939_124	4A	63.1
47252.00	IWB47252	Kukri_c65672_693	4A	63.1
55514.00	IWB55514	RAC875_c23471_298	4A	63.1
4478.00	IWB4478	BobWhite_c8436_391	4A	63.1
71767.00	IWB71767	Tdurum_contig47143_161	4A	63.1
71768.00	IWB71768	Tdurum_contig47143_241	4A	63.1
61748.00	IWB61748	RAC875_rep_c107984_187	4A	63.1
61552.00	IWB61552	RAC875_rep_c106158_701	4A	63.1
50440.00	IWB50440	Kukri_rep_c85536_598	4A	63.1
57528.00	IWB57528	RAC875_c39551_656	4A	63.1
2653.00	IWB2653	BobWhite_c33799_145	4A	63.1
44499.00	IWB44499	Kukri_c35948_758	4A	63.1
59773.00	IWB59773	RAC875_c63420_397	4A	63.1
44537.00	IWB44537	Kukri_c36204_152	4A	63.1
35545.00	IWB35545	IAAV883	4A	63.1
11490.00	IWB11490	BS00087276_51	4A	63.1
11491.00	IWB11491	BS00087277_51	4A	63.1
23864.00	IWB23864	Excalibur_c22830_1989	4A	63.1
28865.00	IWB28865	Excalibur_c780_508	4A	63.1
28866.00	IWB28866	Excalibur_c780_694	4A	63.1
43867.00	IWB43867	Kukri_c3056_4405	4A	63.1

44830.00	IWB44830	Kukri_c3866_718	4A	63.1
72383.00	IWB72383	Tdurum_contig54776_1396	4A	63.1
72384.00	IWB72384	Tdurum_contig54776_1489	4A	63.1
78296.00	IWA4084	wsnp_Ex_c5072_9006966	4A	63.1
78907.00	IWA4858	wsnp_Ex_c8976_14963826	4A	63.1
39523.00	IWB39523	Ku_c4868_489	4A	63.1
78769.00	IWA4690	wsnp_Ex_c7489_12810235	4A	63.1
59560.00	IWB59560	RAC875_c60861_95	4A	63.1
9476.00	IWB9476.1	BS00065188_51	4A	63.1
28864.00	IWB28864.1	Excalibur_c780_400	4A	63.1
22343.00	IWB22343.1	Excalibur_c13953_179	4A	63.1
1512.00	IWB1512.1	BobWhite_c21647_287	4A	63.1
53296.00	IWB53296.1	RAC875_c1126_1769	4A	63.1
65414.00	IWB65414.1	TA001522-0900	4A	63.1
57997.00	IWB57997.1	RAC875_c43902_71	4A	63.1
27531.00	IWB27531.1	Excalibur_c55058_182	4A	63.1
6186.00	IWB6186	BS00010202_51	4A	63.1
24690.00	IWB24690.1	Excalibur_c2827_107	4A	63.1
57683.00	IWB57683	RAC875_c41169_562	4A	63.1
34246.00	IWB34246	GENE-4923_281	4A	63.1
34365.00	IWB34365	IAAV136	4A	63.1
60881.00	IWB60881	RAC875_c8714_653	4A	63.1
21850.00	IWB21850	Excalibur_c113341_139	4A	63.1
30795.00	IWB30795	Excalibur_rep_c66939_849	4A	63.1
22422.00	IWB22422	Excalibur_c1439_880	4A	63.1
12444.00	IWB12444	BS00109911_51	4A	63.1
42399.00	IWB42399	Kukri_c20938_270	4A	63.1
11205.00	IWB11205	BS00081698_51	4A	63.1
31039.00	IWB31039	Excalibur_rep_c69170_425	4A	63.1
47103.00	IWB47103	Kukri_c63845_771	4A	63.1
78908.00	IWA4859	wsnp_Ex_c8976_14964359	4A	63.1
59345.00	IWB59345	RAC875_c58134_104	4A	63.1
23632.00	IWB23632	Excalibur_c21395_1464	4A	63.1
19596.00	IWB19596	Ex_c103537_874	4A	63.1
29737.00	IWB29737	Excalibur_rep_c101560_2243	4A	63.1
9058.00	IWB9058	BS00063433_51	4A	63.1
29950.00	IWB29950	Excalibur_rep_c103537_1054	4A	63.1
30571.00	IWB30571	Excalibur_rep_c112003_621	4A	63.1
39716.00	IWB39716	Ku_c61_424	4A	63.1
1405.00	IWB1405	BobWhite_c20558_209	4A	63.1
36673.00	IWB36673	Jagger_c6192_53	4A	63.1
67740.00	IWB67740	Tdurum_contig12802_393	4A	63.1

Supplementary table 5: Chromosome 2B linkage map developed for the RL6071×Tr129 doubled haploid Subset 2 population lacking resistance to race A (TTKSK)

	**Locus**	**Wheat 90K**	**SNP**	**Chr location**	**CM**	**KASP marker**
1	57369	IWB57369	RAC875_c38003_164	2B	0.0	
2	10434	IWB10434	BS00070050_51	2B	2.2	Kwh599
3	10435	IWB10435	BS00070051_51	2B	2.2	
4	10616	IWB10616	BS00072619_51	2B	2.2	
5	10617	IWB10617	BS00072620_51	2B	2.2	
6	10512	IWB10512	BS00070900_51	2B	3.3	
7	17530	IWB17530	D_F5XZDLF01CFO7W_135	2B	3.3	
8	43883	IWB43883	Kukri_c3067_398	2B	4.4	
9	45508	IWB45508	Kukri_c45103_371	2B	4.4	
10	9206	IWB9206	BS00064153_51	2B	4.4	
11	65267	IWB65267	RFL_Contig772_2612	2B/2D	4.4	
12	39393	IWB39393	Ku_c41677_924	2B	22.5	
13	27391	IWB27391	Excalibur_c53516_119	2B	22.5	
14	65494	IWB65494	TA002079-5951	2B	22.5	
15	78449	IWA4284	wsnp_Ex_c56027_58306755	2B	22.5	
16	23418	IWB23418	Excalibur_c20050_133	2B	22.5	
17	43573	IWB43573	Kukri_c28478_750	2B	22.5	
18	67043	IWB67043	Tdurum_contig11379_1047	2B	22.5	
19	32470	IWB32470	GENE-1421_706	2B	22.5	
20	31706	IWB31706	GENE-0137_469	2B	22.5	
21	43753	IWB43753	Kukri_c29640_212	2B	22.5	
22	43754	IWB43754	Kukri_c29640_92	2B	22.5	
23	67044	IWB67044	Tdurum_contig11379_468	2B	22.5	
24	39233	IWB39233	Ku_c34562_480	2B	22.5	
25	42859	IWB42859	Kukri_c23771_483	2B	22.5	
26	21110	IWB21110	Ex_c67274_1225	2B	22.5	
27	55258	IWB55258	RAC875_c21868_792	2B	22.5	
28	78450	IWA4285	wsnp_Ex_c56027_58308093	2B	22.5	
29	23421	IWB23421	Excalibur_c20050_748	2B	22.5	
30	24406	IWB24406	Excalibur_c26099_106	2B	22.5	

31	32469	IWB32469	GENE-1421_124	2B	22.5	
32	79883	IWA6184	wsnp_JD_c8085_9129899	2B	22.5	
33	55434	IWB55434	RAC875_c22997_534	2B	22.5	
34	49164	IWB49164	Kukri_rep_c106290_204	2B	22.5	
35	8063	IWB8063	BS00036721_51	2B	22.5	
36	28920	IWB28920	Excalibur_c7963_1722	2B	22.5	
37	32471	IWB32471	GENE-1421_802	2B	22.5	
38	31710	IWB31710	GENE-0137_782	2B	22.5	
39	23439	IWB23439	Excalibur_c20196_503	2B	24.8	
40	23438	IWB23438	Excalibur_c20196_264	2B	24.8	
41	61709	IWB61709	RAC875_rep_c107531_66	2B	24.8	
42	67443	IWB67443	Tdurum_contig12095_76	2B	24.8	
43	77007	IWA2391	wsnp_Ex_c19260_28187434	2B	24.8	
44	76198	IWA1359	wsnp_Ex_c10596_17293192	2B	24.8	
45	76199	IWA1360	wsnp_Ex_c10596_17293363	2B	24.8	
46	54531	IWB54531	RAC875_c17720_570	2B	24.8	
47	54530	IWB54530	RAC875_c17720_501	2B	24.8	
48	54529	IWB54529	RAC875_c17720_436	2B	24.8	
49	55506	IWB55506	RAC875_c23425_208	2B	24.8	
50	75619	IWA413	wsnp_BF146221B_Ta_2_2	2B	24.8	
51	64186	IWB64186	RFL_Contig3027_1269	2B	24.8	
52	19719	IWB19719	Ex_c12051_875	2B	24.8	
53	34527	IWB34527	IAAV2277	2B	24.8	
54	34849	IWB34849	IAAV4206	2B	24.8	
55	53589	IWB53589	RAC875_c12766_461	2B	24.8	
56	8280	IWB8280	BS00041585_51	2B	24.8	Kwh598
57	8281	IWB8281	BS00041587_51	2B	24.8	
58	35959	IWB35959	IACX445	2B	24.8	
59	59781	IWB59781	RAC875_c6358_1091	2B	24.8	
60	28247	IWB28247	Excalibur_c63941_83	2B	24.8	
61	1748	IWB1748	BobWhite_c23790_98	2B	24.8	
62	11939	IWB11939	BS00095573_51	2B	24.8	
63	23172	IWB23172	Excalibur_c18630_268	2B	24.8	
64	29273	IWB29273	Excalibur_c89477_207	2B	24.8	
65	34673	IWB34673	IAAV3165	2B	24.8	
66	44675	IWB44675	Kukri_c37311_136	2B	24.8	

67	6117	IWB6117	BS00009807_51	2B	24.8	
68	80066	IWA6474	wsnp_Ku_c12721_20478606	2B	24.8	
69	167	IWB167	BobWhite_c11059_169	2B	24.8	
70	40572	IWB40572	Kukri_c11318_591	2B	24.8	
71	6525	IWB6525	BS00012078_51	2B	24.8	
72	69541	IWB69541	Tdurum_contig28494_288	2B	24.8	
73	9207	IWB9207	BS00064155_51	2B	24.8	
74	46832	IWB46832	Kukri_c59939_94	2B	24.8	
75	32171	IWB32171	GENE-0989_875	2B	24.8	
76	70198	IWB70198	Tdurum_contig31244_181	2B	24.8	
77	46423	IWB46423	Kukri_c54262_436	2B	24.8	
78	23173	IWB23173	Excalibur_c18630_283	2B	24.8	
79	61653	IWB61653	RAC875_rep_c107031_84	2B	24.8	
80	6886	IWB6886	BS00022060_51	2B/5B	24.8	
81	77147	IWA2571	wsnp_Ex_c21092_30220342	2B	29.3	
82	35644	IWB35644	IACX1098	2B	29.3	
83	3178	IWB3178	BobWhite_c41535_52	2B	29.3	
84	77148	IWA2572	wsnp_Ex_c21092_30220702	2B	29.3	
85	46469	IWB46469	Kukri_c5497_312	2B	29.3	
86	29319	IWB29319	Excalibur_c9093_1469	2B	33.7	
87	55936	IWB55936	RAC875_c26469_480	2B	33.7	
88	56961	IWB56961	RAC875_c34516_316	2B	33.7	Kwh596
89	11952	IWB11952	BS00096182_51	2B	33.7	
90	62309	IWB62309	RAC875_rep_c115152_90	2B	49.4	
91	31001	IWB31001	Excalibur_rep_c68899_1400	2B	49.4	
92	32627	IWB32627	GENE-1741_103	2B	49.4	
93	72307	IWB72307	Tdurum_contig53156_111	2B	49.4	
94	60831	IWB60831	RAC875_c86069_65	2B	49.4	
95	36124	IWB36124	IACX6223	2B	49.4	
96	7346	IWB7346	BS00022966_51	2B	49.4	
97	24614	IWB24614	Excalibur_c27716_544	2B	58.2	
98	32327	IWB32327	GENE-1246_417	2B	58.2	
99	64716	IWB64716	RFL_Contig4542_1281	2B	58.2	
100	73543	IWB73543	Tdurum_contig81917_141	2B	58.2	
101	73598	IWB73598	Tdurum_contig82821_324	2B	58.2	
102	70370	IWB70370	Tdurum_contig32621_437	2B/2D	58.2	

103	45820	IWB45820	Kukri_c4790_1137	2B/2D	58.2	
104	39513	IWB39513	Ku_c4790_797	2B	58.2	
105	47380	IWB47380	Kukri_c67276_76	2B	58.2	
106	10568	IWB10568	BS00071690_51	2B	58.2	
107	47379	IWB47379	Kukri_c67276_103	2B	58.2	
108	45822	IWB45822	Kukri_c4790_154	2B	58.2	
109	45825	IWB45825	Kukri_c4790_923	2B	58.2	
110	71006	IWB71006	Tdurum_contig42214_2997	2B	62.5	
111	18439	IWB18439	D_GBF1XID02GVTKX_76	2B	62.5	
112	42992	IWB42992	Kukri_c24586_1089	2B	62.5	
113	55177	IWB55177	RAC875_c21378_474	2B	62.5	
114	42993	IWB42993	Kukri_c24586_923	2B	62.5	
115	63700	IWB63700	RFL_Contig1633_304	2B	63.6	
116	58853	IWB58853	RAC875_c52566_551	2B	63.6	
117	6607	IWB6607	BS00014923_51	2B	63.6	
118	58852	IWB58852	RAC875_c52566_447	2B	63.6	
119	78794	IWA4720	wsnp_Ex_c7738_13195349	2B	63.6	
120	58854	IWB58854	RAC875_c52566_644	2B	63.6	
121	4100	IWB4100	BobWhite_c6166_319	2B	63.6	
122	45644	IWB45644	Kukri_c4632_115	2B	63.6	
123	50839	IWB50839	Ra_c107358_279	2B	63.6	
124	34697	IWB34697	IAAV3305	2B/7A	63.6	
125	12643	IWB12643	BS00111325_51	2B/7D	64.7	Kwh593
126	11568	IWB11568	BS00088575_51	2B	64.7	
127	22202	IWB22202	Excalibur_c1305_662	2B	64.7	
128	32005	IWB32005	GENE-0644_370	2B	64.7	
129	32007	IWB32007	GENE-0644_421	2B	64.7	
130	34545	IWB34545	IAAV2381	2B	64.7	
131	53910	IWB53910	RAC875_c14362_529	2B	64.7	
132	61587	IWB61587	RAC875_rep_c106400_276	2B	64.7	
133	1602	IWB1602	BobWhite_c22503_605	2B	64.7	
134	77647	IWA3257	wsnp_Ex_c30_66389	2B	64.7	
135	24382	IWB24382	Excalibur_c25921_230	2B	64.7	
136	61926	IWB61926	RAC875_rep_c109975_192	2B	64.7	
137	23131	IWB23131	Excalibur_c18417_285	2B	64.7	
138	10805	IWB10805	BS00075410_51	2B	64.7	

139	43295	IWB43295	Kukri_c26474_540	2B	64.7
140	74681	IWB74681	tplb0040e02_1293	2B	64.7
141	41645	IWB41645	Kukri_c16760_1086	2B	64.7
142	41647	IWB41647	Kukri_c16760_259	2B	64.7
143	22020	IWB22020	Excalibur_c12004_73	2B	64.7
144	24397	IWB24397	Excalibur_c26042_260	2B	64.7
145	32006	IWB32006	GENE-0644_42	2B	64.7
146	62221	IWB62221	RAC875_rep_c113555_122	2B	64.7
147	62870	IWB62870	RAC875_rep_c72435_90	2B	64.7
148	22543	IWB22543	Excalibur_c15031_73	2B	64.7
149	32009	IWB32009	GENE-0652_360	2B	64.7
150	74034	IWB74034	Tdurum_contig98206_211	2B	64.7
151	44605	IWB44605	Kukri_c36756_316	2B	64.7
152	61973	IWB61973	RAC875_rep_c110493_512	2B	64.7
153	73607	IWB73607	Tdurum_contig83066_276	2B	64.7
154	79320	IWA5377	wsnp_Ex_rep_c67257_65786614	2B	64.7
155	75564	IWA328	wsnp_BE499478B_Ta_2_1	2B	64.7
156	78174	IWA3924	wsnp_Ex_c45094_50985067	2B	64.7
157	65801	IWB65801	TA004199-0831	2B	64.7
158	22235	IWB22235	Excalibur_c13239_687	2B	64.7
159	78732	IWA4642	wsnp_Ex_c7246_12443506	2B	64.7
160	19714	IWB19714	Ex_c12004_1006	2B	64.7
161	37419	IWB37419	JD_c39990_130	2B	64.7
162	40383	IWB40383	Kukri_c106282_114	2B	64.7
163	78480	IWA4323	wsnp_Ex_c57_116914	2B	64.7
164	80030	IWA6427	wsnp_Ku_c11665_18999583	2B	64.7
165	47487	IWB47487	Kukri_c6973_344	2B	64.7
166	40021	IWB40021	Ku_c7740_879	2B	64.7
167	44610	IWB44610	Kukri_c36783_91	2B	64.7
168	8214	IWB8214	BS00040251_51	2B	64.7
169	10669	IWB10669	BS00073426_51	2B	64.7
170	32069	IWB32069	GENE-0724_612	2B	64.7
171	29391	IWB29391	Excalibur_c9248_771	2B	64.7
172	30179	IWB30179	Excalibur_rep_c106124_239	2B	64.7
173	32441	IWB32441	GENE-1389_59	2B	64.7
174	35747	IWB35747	IACX1412	2B	64.7

175	42613	IWB42613	Kukri_c22200_1171	2B	64.7
176	75339	IWA10	wsnp_BE399688B_Ta_2_1	2B	64.7
177	78933	IWA4894	wsnp_Ex_c9248_15372536	2B	64.7
178	11751	IWB11751	BS00092275_51	2B	64.7
179	21988	IWB21988	Excalibur_c11863_289	2B	64.7
180	37811	IWB37811	JD_c767_567	2B	64.7
181	53512	IWB53512	RAC875_c1226_652	2B	64.7
182	5274	IWB5274	BobWhite_rep_c61602_139	2B	64.7
183	8834	IWB8834	BS00061268_51	2B	64.7
184	27810	IWB27810	Excalibur_c584_726	2B	64.7
185	7755	IWB7755	BS00030090_51	2B	64.7
186	11725	IWB11725	BS00091671_51	2B	64.7
187	23529	IWB23529	Excalibur_c20647_643	2B	64.7
188	59779	IWB59779	RAC875_c63545_85	2B	64.7
189	42648	IWB42648	Kukri_c22419_541	2B	64.7
190	12642	IWB12642	BS00111324_51	2B/7D	64.7
191	8772	IWB8772	BS00059315_51	2B	64.7
192	29853	IWB29853	Excalibur_rep_c102657_575	2B	64.7
193	60585	IWB60585	RAC875_c79857_301	2B	64.7
194	12041	IWB12041	BS00098490_51	2B	64.7
195	62112	IWB62112	RAC875_rep_c112008_519	2B	64.7
196	38861	IWB38861	Ku_c23446_2209	2B	64.7
197	9690	IWB9690	BS00065994_51	2B	64.7
198	9689	IWB9689	BS00065993_51	2B	64.7
199	25795	IWB25795	Excalibur_c37239_916	2B	64.7
200	28615	IWB28615	Excalibur_c721_484	2B	64.7
201	44515	IWB44515	Kukri_c36026_68	2B	64.7
202	55876	IWB55876	RAC875_c2600_188	2B	64.7
203	22236	IWB22236	Excalibur_c13239_85	2B	64.7
204	8922	IWB8922	BS00062774_51	2B/2D	64.7
205	41646	IWB41646	Kukri_c16760_1427	2B	64.7
206	12187	IWB12187	BS00102480_51	2B	64.7
207	58034	IWB58034	RAC875_c4430_2213	2B	64.7
208	59183	IWB59183	RAC875_c5638_1010	2B	64.7
209	11750	IWB11750	BS00092273_51	2B	64.7
210	32439	IWB32439	GENE-1389_396	2B	64.7

211	32440	IWB32440	GENE-1389_458	2B	64.7	
212	36599	IWB36599	Jagger_c4412_103	2B	64.7	
213	36600	IWB36600	Jagger_c4412_265	2B	64.7	
214	36153	IWB36153	IACX6411	2B	64.7	
215	57258	IWB57258	RAC875_c37191_584	2B/2D	64.7	
216	7198	IWB7198	BS00022657_51	2B	64.7	
217	8835	IWB8835	BS00061269_51	2B	64.7	
218	13310	IWB13310	CAP12_c2565_239	2B	64.7	
219	27246	IWB27246	Excalibur_c5169_598	2B	64.7	
220	3522	IWB3522	BobWhite_c4661_500	2B	64.7	
221	36708	IWB36708	Jagger_c6853_60	2B	68.0	Kwh592
222	8535	IWB8535	BS00049378_51	2B/5B	72.5	
223	27601	IWB27601	Excalibur_c55781_129	2B/5B	72.5	
224	8536	IWB8536	BS00049380_51	2B/5B	72.5	
225	42187	IWB42187	Kukri_c19751_873	2B	72.5	
226	73264	IWB73264	Tdurum_contig7529_227	2B	72.5	
227	52584	IWB52584	Ra_c72477_2165	2B	72.5	
228	50388	IWB50388	Kukri_rep_c80379_398	2B	72.5	
229	26404	IWB26404	Excalibur_c43037_831	2B	72.5	
230	38293	IWB38293	Ku_c12037_482	2B	72.5	
231	53996	IWB53996	RAC875_c14775_1048	2B	72.5	
232	75491	IWA226	wsnp_BE490267A_Ta_2_1	2B	72.5	
233	43186	IWB43186	Kukri_c25815_263	2B	72.5	
234	11849	IWB11849	BS00093993_51	2B	72.5	
235	76157	IWA1305	wsnp_Ex_c10251_16815792	2B/7B	72.5	Kwh588
236	36605	IWB36605	Jagger_c4502_69	2B	72.5	
237	57202	IWB57202	RAC875_c36614_344	2B	72.5	
238	20275	IWB20275	Ex_c2510_477	2B	72.5	
239	50067	IWB50067	Kukri_rep_c70870_919	2B	72.5	
240	13303	IWB13303	CAP12_c2472_60	2B	72.5	
241	61287	IWB61287	RAC875_c9932_705	2B	72.5	
242	56572	IWB56572	RAC875_c31162_706	2B	72.5	
243	52361	IWB52361	Ra_c6367_387	2B	72.5	
244	43954	IWB43954	Kukri_c31165_835	2B	72.5	
245	44123	IWB44123	Kukri_c32700_104	2B	72.5	
246	36041	IWB36041	IACX5850	2B	72.5	Kwh589

247	34989	IWB34989	IAAV5226	2B	72.5	
248	80048	IWA6453	wsnp_Ku_c12037_19549078	2B	72.5	Kwh572
249	77479	IWA3037	wsnp_Ex_c26281_35525999	2B	72.5	
250	77478	IWA3035	wsnp_Ex_c26281_35525243	2B	72.5	
251	76863	IWA2189	wsnp_Ex_c17127_25756019	2B	72.5	
252	70131	IWB70131	Tdurum_contig30930_184	2B	72.5	
253	79347	IWA5411	wsnp_Ex_rep_c67543_66165372	2B	72.5	
254	7263	IWB7263	BS00022800_51	2B	72.5	
255	38254	IWB38254	Ku_c11652_1720	2B	72.5	
256	64817	IWB64817	RFL_Contig4856_71	2B	72.5	Kwh590
257	76156	IWA1304	wsnp_Ex_c10251_16815404	2B	72.5	
258	76223	IWA1393	wsnp_Ex_c10838_17631243	2B	72.5	
259	30854	IWB30854	Excalibur_rep_c67411_210	2B	73.6	
260	34696	IWB34696	IAAV3303	2B	73.6	
261	50859	IWB50859	Ra_c108387_863	2B	73.6	
262	60039	IWB60039	RAC875_c66657_91	2B	73.6	
263	21140	IWB21140	Ex_c68194_1994	2B	73.6	
264	73040	IWB73040	Tdurum_contig66317_77	2B	73.6	
265	51104	IWB51104	Ra_c14316_850	2B	73.6	
266	53902	IWB53902	RAC875_c14316_584	2B	73.6	
267	60374	IWB60374	RAC875_c7540_366	2B	73.6	
268	59086	IWB59086	RAC875_c54947_108	2B	73.6	
269	49862	IWB49862	Kukri_rep_c69169_334	2B	73.6	
270	49783	IWB49783	Kukri_rep_c68360_1476	2B	73.6	
271	48280	IWB48280	Kukri_c920_319	2B	73.6	
272	48279	IWB48279	Kukri_c920_1379	2B	73.6	
273	48278	IWB48278	Kukri_c920_128	2B	73.6	
274	47243	IWB47243	Kukri_c6552_4243	2B	73.6	
275	40823	IWB40823	Kukri_c12442_454	2B	73.6	
276	35867	IWB35867	IACX2626	2B	73.6	
277	28487	IWB28487	Excalibur_c6807_464	2B	73.6	
278	28486	IWB28486	Excalibur_c6807_1155	2B	73.6	
279	27166	IWB27166	Excalibur_c5064_765	2B	73.6	
280	27165	IWB27165	Excalibur_c5064_284	2B	73.6	
281	23356	IWB23356	Excalibur_c19649_1500	2B	73.6	
282	21199	IWB21199	Ex_c69493_1208	2B	73.6	

283	21139	IWB21139	Ex_c68194_1532	2B	73.6	
284	43015	IWB43015	Kukri_c24669_1235	2B	73.6	
285	70826	IWB70826	Tdurum_contig42095_3235	2B	73.6	
286	76898	IWA2237	wsnp_Ex_c1758_3326792	2B	73.6	
287	81070	IWA7825	wsnp_Ra_c28955_38371323	2B	73.6	
288	62675	IWB62675	RAC875_rep_c70342_1644	2B	73.6	
289	43016	IWB43016	Kukri_c24669_51	2B	73.6	
290	73022	IWB73022	Tdurum_contig65349_140	2B	73.6	
291	60373	IWB60373	RAC875_c7540_118	2B	73.6	
292	79422	IWA5513	wsnp_Ex_rep_c68194_66973531	2B	73.6	
293	79333	IWA5397	wsnp_Ex_rep_c67411_65994109	2B	73.6	
294	79421	IWA5512	wsnp_Ex_rep_c68194_66973114	2B	73.6	
295	78431	IWA4256	wsnp_Ex_c54998_57670603	2B	73.6	
296	55916	IWB55916	RAC875_c26278_459	2B	73.6	
297	37941	IWB37941	JG_c2092_196	2B	73.6	
298	53595	IWB53595	RAC875_c12803_916	2B	73.6	
299	46616	IWB46616	Kukri_c56872_402	2B	73.6	
300	70825	IWB70825	Tdurum_contig42095_2454	2B	73.6	
301	29225	IWB29225	Excalibur_c88037_341	2B	73.6	
302	22798	IWB22798	Excalibur_c16532_499	2B	73.6	
303	25132	IWB25132	Excalibur_c31737_169	2B	73.6	
304	10411	IWB10411	BS00069685_51	2B	74.7	
305	79813	IWA6076	wsnp_JD_c47318_32176833	2B	75.9	Kwh574
306	45296	IWB45296	Kukri_c4294_371	2B	75.9	Kwh570
307	27578	IWB27578	Excalibur_c5557_201	2B	77.0	
308	31274	IWB31274	Excalibur_rep_c77221_93	2B	77.0	
309	29491	IWB29491	Excalibur_c94980_51	2B	77.0	
310	28337	IWB28337	Excalibur_c64983_65	2B	77.0	Kwh576
311	23831	IWB23831	Excalibur_c22637_235	2B/2D	77.0	
312	102	IWB102	BobWhite_c1072_100	2B	77.0	
313	2452	IWB2452	BobWhite_c3146_128	2B	77.0	
314	11652	IWB11652	BS00090235_51	2B	77.0	
315	45055	IWB45055	Kukri_c40719_491	2B	77.0	
316	68759	IWB68759	Tdurum_contig18858_324	2B	77.0	
317	7403	IWB7403	BS00023060_51	2B	77.0	
318	7226	IWB7226	BS00022717_51	2B	77.0	

319	5666	IWB5666	BobWhite_s64174_145	2B	77.0	
320	47198	IWB47198	Kukri_c64930_353	2B	77.0	
321	31015	IWB31015	Excalibur_rep_c68985_110	2B	77.0	
322	67207	IWB67207	Tdurum_contig11653_370	2B	77.0	
323	13168	IWB13168	CAP12_c1037_120	2B	77.0	
324	67208	IWB67208	Tdurum_contig11653_371	2B	77.0	
325	11092	IWB11092	BS00079621_51	2B	77.0	Kwh575
326	60488	IWB60488	RAC875_c77816_365	2Dx	77.0	
327	25842	IWB25842	Excalibur_c3762_829	2B/6B	77.0	
328	45652	IWB45652	Kukri_c46361_295	2B	77.0	
329	73241	IWB73241	Tdurum_contig74826_330	2B	77.0	
330	8717	IWB8717	BS00057475_51	2B	77.0	
331	32221	IWB32221	GENE-1080_39	2B	77.0	
332	25841	IWB25841	Excalibur_c3762_715	2B	77.0	
333	28850	IWB28850	Excalibur_c7755_1321	2B	77.0	
334	28849	IWB28849	Excalibur_c7755_1057	2B	77.0	
335	4501	IWB4501	BobWhite_c8664_94	2B	77.0	
336	36229	IWB36229	IACX7718	2B	77.0	
337	12298	IWB12298	BS00106901_51	2B	79.2	
338	43278	IWB43278	Kukri_c26311_891	2B	79.2	
339	9117	IWB9117	BS00063727_51	2B	79.2	
340	48248	IWB48248	Kukri_c9118_1774	2B	79.2	
341	32189	IWB32189	GENE-1012_303	2B	79.2	
342	43934	IWB43934	Kukri_c31059_130	2B	79.2	
343	60406	IWB60406	RAC875_c76186_353	2B	79.2	
344	13421	IWB13421	CAP12_c4899_191	2B	79.2	
345	39379	IWB39379	Ku_c40808_718	2B	79.2	
346	13162	IWB13162	CAP11_s8679_211	2B	79.2	
347	9247	IWB9247	BS00064318_51	2B	79.2	
348	6453	IWB6453	BS00011630_51	2B	79.2	
349	1190	IWB1190	BobWhite_c18540_97	2B	79.2	
350	1188	IWB1188	BobWhite_c18540_351	2B	79.2	
351	54520	IWB54520	RAC875_c17658_1419	2B	79.2	
352	32190	IWB32190	GENE-1012_680	2B	79.2	
353	10081	IWB10081	BS00067572_51	2B	79.2	
354	30560	IWB30560	Excalibur_rep_c111801_55	2B	79.2	

355	43169	IWB43169	Kukri_c25716_284	2B	79.2	
356	15068	IWB15068	CAP8_rep_c8162_101	2B	79.2	
357	37862	IWB37862	JD_c8871_331	2B	79.2	
358	8789	IWB8789	BS00059896_51	2B	79.2	
359	81194	IWA7996	wsnp_Ra_c54256_57628288	2B	79.2	
360	77103	IWA2514	wsnp_Ex_c20182_29230528	2B	79.2	
361	9248	IWB9248	BS00064322_51	2B	79.2	
362	81334	IWA8195	wsnp_Ra_rep_c74497_72390803	2B	79.2	
363	81396	IWA8295	wsnp_RFL_Contig2123_1397739	2B	79.2	
364	21691	IWB21691	Excalibur_c10634_156	2B	79.2	
365	78034	IWA3742	wsnp_Ex_c40976_47910672	2B	79.2	
366	51068	IWB51068	Ra_c13679_2284	2B	79.2	Kwh577
367	40155	IWB40155	Ku_c9369_1726	2Dx	79.2	
368	28807	IWB28807	Excalibur_c76665_98	2B	79.2	
369	1630	IWB1630	BobWhite_c22728_257	2B	79.2	
370	46242	IWB46242	Kukri_c52356_96	2B	79.2	
371	78033	IWA3741	wsnp_Ex_c40976_47910144	2B	79.2	
372	64008	IWB64008	RFL_Contig2612_826	2B	79.2	
373	76474	IWA1708	wsnp_Ex_c12922_20473104	2B	79.2	
374	78457	IWA4294	wsnp_Ex_c5619_9884202	2B	79.2	
375	60670	IWB60670	RAC875_c8197_810	2B	79.2	
376	4571	IWB4571	BobWhite_c908_64	2B	79.2	
377	61802	IWB61802	RAC875_rep_c108548_163	2B/2D	79.2	
378	74910	IWB74910	tplb0048g05_866	2B	79.2	
379	68283	IWB68283	Tdurum_contig14707_251	2B	79.2	
380	79841	IWA6122	wsnp_JD_c6010_7167159	2B	79.2	
381	65989	IWB65989	TA005601-0686	2B	79.2	
382	55526	IWB55526	RAC875_c23538_1517	2B	79.2	
383	26191	IWB26191	Excalibur_c40976_87	2B	79.2	
384	26189	IWB26189	Excalibur_c40976_111	2B	79.2	
385	1631	IWB1631	BobWhite_c22728_78	2B	79.2	
386	318	IWB318	BobWhite_c12157_747	2B	79.2	
387		IWA7659	wsnp_Ra_c16333_24961476	2B	80.3	Kwh28
388	2286	IWB2286	BobWhite_c29596_649	2B	82.6	
389	8386	IWB8386	BS00044983_51	2B	82.6	
390	28962	IWB28962	Excalibur_c80601_308	2B	82.6	

391	75246	IWB75246	tplb0060d11_1137	2B	82.6	
392	34664	IWB34664	IAAV3100	2B	82.6	
393	28963	IWB28963	Excalibur_c80601_83	2B	82.6	
394	36072	IWB36072	IACX5941	2B	82.6	Kwh578
395	2943	IWB2943	BobWhite_c38001_528	2B	82.6	
396	77576	IWA3176	wsnp_Ex_c28627_37743031	2B	82.6	Kwh32
397	75247	IWB75247	tplb0060d11_435	2B	82.6	
398	77358	IWA2873	wsnp_Ex_c24135_33382521	2B	82.6	
399	77359	IWA2874	wsnp_Ex_c24135_33382700	2B	82.6	
400	73211	IWB73211	Tdurum_contig72054_94	2B	82.6	
401	2593	IWB2593	BobWhite_c32938_264	2B	82.6	
402	74201	IWB74201	tplb0024l03_910	2B	82.6	
403	80132	IWA6561	wsnp_Ku_c15498_24122936	2B	82.6	
404	44545	IWB44545	Kukri_c36266_120	2B	82.6	
405	56400	IWB56400	RAC875_c29937_325	2B	82.6	
406	26416	IWB26416	Excalibur_c43102_132	2B	82.6	
407	25544	IWB25544	Excalibur_c3506_610	2B	82.6	
408	24230	IWB24230	Excalibur_c2484_2113	2B	83.7	
409	58747	IWB58747	RAC875_c51420_237	2B/2D	84.8	
410		IWA2215	wsnp_Ex_c174_340829		84.8	Kwh29
411	14677	IWB14677	CAP8_c3234_216	2B/2D	85.9	
412	59956	IWB59956	RAC875_c65495_424	2B/2D	85.9	
413	23010	IWB23010	Excalibur_c17745_493	2B/2D	85.9	
414	10251	IWB10251	BS00068181_51	2B	85.9	
415	11888	IWB11888	BS00094578_51	2B	85.9	
416	60041	IWB60041	RAC875_c66695_560	2B	85.9	
417	79641	IWA5809	wsnp_JD_c12346_12606967	2B	85.9	
418	80550	IWA7112	wsnp_Ku_c48694_54811376	2B	85.9	
419	39525	IWB39525	Ku_c48694_1284	2B	85.9	
420	79078	IWA5081	wsnp_Ex_rep_c103064_88104690	2B	85.9	
421	80551	IWA7113	wsnp_Ku_c48694_54811423	2B	85.9	
422	72654	IWB72654	Tdurum_contig59546_695	2B	85.9	
423	54357	IWB54357	RAC875_c16752_283	2B	85.9	
424	7265	IWB7265	BS00022805_51	2B	85.9	
425	43633	IWB43633	Kukri_c29052_75	2B	85.9	
426	4648	IWB4648	BobWhite_c9690_94	2B	85.9	

427	9514	IWB9514	BS00065327_51	2B	85.9
428	39394	IWB39394	Ku_c4168_1399	2B	85.9
429	63201	IWB63201	RAC875_rep_c83093_126	2B/2D	85.9
430	69628	IWB69628	Tdurum_contig28795_219	2B	85.9
431	25868	IWB25868	Excalibur_c37753_655	2B	85.9
432	46631	IWB46631	Kukri_c57014_168	2B	85.9
433	49829	IWB49829	Kukri_rep_c68903_708	2B	85.9
434	29532	IWB29532	Excalibur_c95944_641	2B	85.9
435	69431	IWB69431	Tdurum_contig28113_579	2B/2D	85.9
436	27746	IWB27746	Excalibur_c57713_81	2B	85.9
437	25546	IWB25546	Excalibur_c35074_257	2B	85.9
438	48386	IWB48386	Kukri_c9478_2764	2B/2D	85.9
439	25281	IWB25281	Excalibur_c32980_169	2B/2D	85.9
440	7223	IWB7223	BS00022713_51	2B/2D	85.9
441	49827	IWB49827	Kukri_rep_c68903_159	2B/2D	85.9
442	25869	IWB25869	Excalibur_c37753_754	2B	85.9
443	23660	IWB23660	Excalibur_c2157_173	2B	85.9
444	44694	IWB44694	Kukri_c37462_459	2B	85.9
445	73407	IWB73407	Tdurum_contig77036_338	2B	85.9
446	69631	IWB69631	Tdurum_contig28795_381	2B	85.9
447	73196	IWB73196	Tdurum_contig71365_233	2B	85.9
448	25313	IWB25313	Excalibur_c33221_681	2B	85.9
449	516	IWB516	BobWhite_c13525_262	2B	85.9
450	49828	IWB49828	Kukri_rep_c68903_301	2B	85.9
451	22381	IWB22381	Excalibur_c14201_111	2B	85.9
452	51053	IWB51053	Ra_c13298_783	2B	85.9
453	58646	IWB58646	RAC875_c50422_299	2B/2D	85.9
454	9560	IWB9560	BS00065471_51	2B	85.9
455	5957	IWB5957	BS00004224_51	2B	85.9
456	27744	IWB27744	Excalibur_c57713_105	2B	85.9
457	73846	IWB73846	Tdurum_contig93103_284	2B	85.9
458	73567	IWB73567	Tdurum_contig82393_581	2B	85.9
459	73057	IWB73057	Tdurum_contig67254_504	2B	85.9
460	68692	IWB68692	Tdurum_contig17826_338	2B	85.9
461	19788	IWB19788	Ex_c13213_2992	2B	85.9
462	19787	IWB19787	Ex_c13213_2594	2B	85.9

463	76570	IWA1821	wsnp_Ex_c13865_21720307	2B/2D	85.9	
464	81534	IWA8534	wsnp_RFL_Contig3917_4326857	2B	85.9	
465	41823	IWB41823	Kukri_c17832_1029	2B	85.9	
466	71742	IWB71742	Tdurum_contig47_185	2B	85.9	
467	74179	IWB74179	tplb0024e19_1029	2B	85.9	
468	71741	IWB71741	Tdurum_contig47_148	2B	85.9	
469	9328	IWB9328	BS00064607_51	2B	85.9	
470	54447	IWB54447	RAC875_c17331_129	2B	85.9	
471	77821	IWA3478	wsnp_Ex_c34419_42734849	2B/2D	87.0	
472	26300	IWB26300	Excalibur_c42146_266	2B	87.0	Kwh580
473	38059	IWB38059	Ku_c102096_268	2B	87.0	
474	32354	IWB32354	GENE-1280_188	2B	87.0	
475	10846	IWB10846	BS00076003_51	2B	87.0	Kwh579
476	10845	IWB10845	BS00076000_51	2B	87.0	
477	7514	IWB7514	BS00023902_51	2B	87.0	
478	60866	IWB60866	RAC875_c8684_1668	2Dx	87.0	
479	34617	IWB34617	IAAV283	2B	89.2	
480	11174	IWB11174	BS00081189_51	2B/2D	89.2	
481	10162	IWB10162	BS00067828_51	2B	90.4	
482	25863	IWB25863	Excalibur_c37727_66	2B/2D	90.4	
483	12574	IWB12574	BS00110611_51	2B	90.4	
484	10455	IWB10455	BS00070301_51	2B	108.3	
485	28108	IWB28108	Excalibur_c62234_105	2B	108.3	
486	36334	IWB36334	IACX8947	2B	108.3	
487	32417	IWB32417	GENE-1355_265	2B	108.3	
488	23797	IWB23797	Excalibur_c224_1383	2B	108.3	
489	28191	IWB28191	Excalibur_c63327_110	2B	108.3	
490	11366	IWB11366	BS00084417_51	2B	108.3	
491	32416	IWB32416	GENE-1355_130	2B	108.3	
492	76172	IWA1324	wsnp_Ex_c10441_17078853	2B	108.3	
493	65636	IWB65636	TA003025-1611	2B	108.3	
494	59983	IWB59983	RAC875_c65882_668	2B	108.3	
495	120	IWB120	BobWhite_c10864_436	2B	108.3	
496	22835	IWB22835	Excalibur_c16679_215	2B	108.3	
497	11123	IWB11123	BS00080318_51	2B	108.3	
498	28651	IWB28651	Excalibur_c73027_267	2B	108.3	

499	2534	IWB2534	BobWhite_c32319_268	2B	110.6
500	73380	IWB73380	Tdurum_contig76550_500	2B	110.6
501	52360	IWB52360	Ra_c6355_647	2B	110.6
502	46445	IWB46445	Kukri_c54653_270	2B/5A	110.6
503	34542	IWB34542	IAAV237	2B	110.6
504	33668	IWB33668	GENE-3921_131	2B	110.6
505	36223	IWB36223	IACX7683	2B	110.6
506	33669	IWB33669	GENE-3921_24	2B	110.6
507	44343	IWB44343	Kukri_c34553_188	2B	110.6
508	28561	IWB28561	Excalibur_c7051_1027	2B	110.6
509	8894	IWB8894	BS00062699_51	2B	110.6
510	6107	IWB6107	BS00009763_51	2B	110.6
511	55345	IWB55345	RAC875_c22463_416	2B	110.6
512	3749	IWB3749	BobWhite_c5191_362	2B	110.6
513	30535	IWB30535	Excalibur_rep_c111477_175	2B	110.6
514	2535	IWB2535	BobWhite_c32319_313	2B	110.6
515	8607	IWB8607	BS00051965_51	2B	110.6
516	6113	IWB6113	BS00009791_51	2B	110.6
517	25152	IWB25152	Excalibur_c31864_713	2B	110.6
518	25008	IWB25008	Excalibur_c30729_152	2B	110.6
519	1770	IWB1770	BobWhite_c23950_203	2B	110.6
520	13585	IWB13585	CAP12_rep_c3989_324	2B	110.6
521	7380	IWB7380	BS00023025_51	2B/3D	110.6
522	64863	IWB64863	RFL_Contig5005_761	2B/2D	110.6
523	25016	IWB25016	Excalibur_c30744_181	2B	110.6
524	61339	IWB61339	RAC875_rep_c102485_468	2B	110.6
525	26482	IWB26482	Excalibur_c4372_262	2B	110.6
526	57295	IWB57295	RAC875_c37540_583	2B	110.6
527	55346	IWB55346	RAC875_c22463_494	2B	110.6
528	55615	IWB55615	RAC875_c24205_110	2B	110.6
529	76795	IWA2104	wsnp_Ex_c16144_24583060	2B/3D	110.6
530	69000	IWB69000	Tdurum_contig234_843	2B	110.6
531	8813	IWB8813	BS00060618_51	2B	110.6
532	6379	IWB6379	BS00011276_51	2B	110.6
533	6130	IWB6130	BS00009882_51	2B	110.6
534	58126	IWB58126	RAC875_c45209_331	2B	110.6

535	42028	IWB42028	Kukri_c18754_67	2B	110.6
536	10107	IWB10107	BS00067641_51	2B	110.6
537	43525	IWB43525	Kukri_c28077_282	2B/5A	110.6
538	72571	IWB72571	Tdurum_contig57254_254	2B/4B	110.6
539	28921	IWB28921	Excalibur_c7964_1290	2B/4B	110.6
540	1663	IWB1663	BobWhite_c23052_196	2B/3B	110.6
541	6334	IWB6334	BS00011047_51	2B/2D	110.6
542	5839	IWB5839	BS00003589_51	2B	110.6
543	36009	IWB36009	IACX5726	2B	110.6
544	47254	IWB47254	Kukri_c657_1514	2B	110.6
545	23686	IWB23686	Excalibur_c2174_1681	2B	110.6
546	22586	IWB22586	Excalibur_c15242_306	2B	110.6
547	40501	IWB40501	Kukri_c11040_787	2B	110.6
548	34773	IWB34773	IAAV3800	2B	110.6
549	61956	IWB61956	RAC875_rep_c110344_370	2B	110.6
550	2628	IWB2628	BobWhite_c33464_133	2B	110.6
551	34394	IWB34394	IAAV1502	2B	110.6
552	54597	IWB54597	RAC875_c18063_771	2B	110.6
553	36295	IWB36295	IACX8386	2B	110.6
554	64461	IWB64461	RFL_Contig3713_280	2B	110.6
555	64463	IWB64463	RFL_Contig3713_538	2B	110.6
556	54212	IWB54212	RAC875_c16064_217	2B	110.6
557	43464	IWB43464	Kukri_c27631_1329	2B	110.6
558	28565	IWB28565	Excalibur_c7051_590	2B	110.6
559	28564	IWB28564	Excalibur_c7051_537	2B	110.6
560	1769	IWB1769	BobWhite_c23950_145	2B	110.6
561	9088	IWB9088	BS00063589_51	2B	110.6
562	51555	IWB51555	Ra_c23972_327	2B	110.6
563	8430	IWB8430	BS00046601_51	2B	110.6
564	6223	IWB6223	BS00010438_51	2B	110.6
565	61824	IWB61824	RAC875_rep_c108836_55	2B	110.6
566	61239	IWB61239	RAC875_c98387_145	2B	110.6
567	5976	IWB5976	BS00004405_51	2B	110.6
568	59281	IWB59281	RAC875_c57353_245	2B	110.6
569	5864	IWB5864	BS00003673_51	2B	110.6
570	56526	IWB56526	RAC875_c30797_179	2B	110.6

571	55806	IWB55806	RAC875_c25512_888	2B	110.6
572	54596	IWB54596	RAC875_c18063_665	2B	110.6
573	54594	IWB54594	RAC875_c18063_497	2B	110.6
574	46716	IWB46716	Kukri_c58096_480	2B	110.6
575	44342	IWB44342	Kukri_c34553_110	2B	110.6
576	43052	IWB43052	Kukri_c24939_378	2B	110.6
577	41498	IWB41498	Kukri_c15985_1277	2B	110.6
578	3750	IWB3750	BobWhite_c5191_563	2B	110.6
579	36753	IWB36753	Jagger_c7688_98	2B	110.6
580	36642	IWB36642	Jagger_c5526_105	2B	110.6
581	20875	IWB20875	Ex_c52711_584	2B	110.6
582	46560	IWB46560	Kukri_c55909_1109	2B	110.6
583	54701	IWB54701	RAC875_c1858_2693	2B	110.6
584	59661	IWB59661	RAC875_c62155_156	2B	110.6
585	61988	IWB61988	RAC875_rep_c110690_104	2B	110.6
586	64515	IWB64515	RFL_Contig385_761	2B	110.6
587	61112	IWB61112	RAC875_c9500_1796	2B	110.6
588	36279	IWB36279	IACX8202	2B	110.6
589	36243	IWB36243	IACX7803	2B	110.6
590	29809	IWB29809	Excalibur_rep_c102228_810	2B	110.6
591	28375	IWB28375	Excalibur_c65466_714	2B	110.6
592	25151	IWB25151	Excalibur_c31864_179	2B	110.6
593	25015	IWB25015	Excalibur_c30744_1195	2B	110.6
594	24984	IWB24984	Excalibur_c30571_176	2B	110.6
595	23209	IWB23209	Excalibur_c1883_340	2B	110.6
596	1710	IWB1710	BobWhite_c23443_337	2B	110.6
597	13680	IWB13680	CAP12_s9114_61	2B	110.6
598	64675	IWB64675	RFL_Contig4423_529	2B	110.6
599	64462	IWB64462	RFL_Contig3713_316	2B	110.6
600	6194	IWB6194	BS00010264_51	2B	110.6
601	56596	IWB56596	RAC875_c31328_381	2B	110.6
602	36327	IWB36327	IACX8828	2B	110.6
603	36158	IWB36158	IACX6480	2B	110.6
604	13584	IWB13584	CAP12_rep_c3989_239	2B	110.6
605	12294	IWB12294	BS00106695_51	2B	110.6
606	10418	IWB10418	BS00069756_51	2B	110.6

607	10417	IWB10417	BS00069754_51	2B	110.6	
608	10706	IWB10706	BS00074091_51	2B	110.6	
609	35156	IWB35156	IAAV6151	2B	110.6	
610	42430	IWB42430	Kukri_c21087_79	2B	110.6	
611	43166	IWB43166	Kukri_c25702_948	2B	110.6	
612	11101	IWB11101	BS00079941_51	2B	110.6	
613	40742	IWB40742	Kukri_c12117_134	2B	110.6	
614	36286	IWB36286	IACX8298	2B	110.6	
615	54348	IWB54348	RAC875_c16727_1826	2B	111.7	
616	27661	IWB27661	Excalibur_c56550_71	2B	111.7	
617	5438	IWB5438	BobWhite_rep_c64068_213	2B	111.7	
618	62356	IWB62356	RAC875_rep_c116263_97	2B	111.7	
619	40458	IWB40458	Kukri_c10869_489	2B	111.7	
620	40456	IWB40456	Kukri_c10869_1591	2B	111.7	
621	40455	IWB40455	Kukri_c10869_1366	2B	111.7	Kwh587
622	27660	IWB27660	Excalibur_c56550_425	2B	111.7	
623	9919	IWB9919	BS00066949_51	2B	111.7	
624	5439	IWB5439	BobWhite_rep_c64068_241	2B	111.7	
625	64039	IWB64039	RFL_Contig2751_1562	2B	111.7	
626	40514	IWB40514	Kukri_c11099_1033	2B	111.7	
627	50532	IWB50532	Kukri_rep_c95118_467	2B	121.6	
628	49462	IWB49462	Kukri_rep_c110602_283	2B	131.5	
629	10173	IWB10173	BS00067878_51	2B	131.5	Kwh586
630	5978	IWB5978	BS00004413_51	2B	131.5	
631	77643	IWA3252	wsnp_Ex_c298_580660	2B	132.6	Kwh585
632	24255	IWB24255	Excalibur_c25043_357	2B	140.4	
633	11171	IWB11171	BS00081138_51	2B	140.4	
634	54548	IWB54548	RAC875_c17798_66	2B	140.4	
635	60398	IWB60398	RAC875_c7602_1205	2B	140.4	
636	48531	IWB48531	Kukri_c9898_1766	2B	140.4	
637	29461	IWB29461	Excalibur_c94383_157	2B	140.4	
638	36519	IWB36519	Jagger_c2989_134	2B	140.4	
639	43915	IWB43915	Kukri_c30917_250	2B	140.4	
640	2181	IWB2181	BobWhite_c2822_228	2B	140.4	
641	54766	IWB54766	RAC875_c19042_2102	2B	140.4	
642	12118	IWB12118	BS00100118_51	2B	140.4	Kwh583

643	3970	IWB3970	BobWhite_c5756_532	2B	140.4
644	34450	IWB34450	IAAV1798	2B	140.4
645	28691	IWB28691	Excalibur_c73791_215	2B	140.4
646	48533	IWB48533	Kukri_c9898_328	2B	140.4
647	44399	IWB44399	Kukri_c3501_1175	2B	140.4
648	48532	IWB48532	Kukri_c9898_2063	2B	140.4
649	24806	IWB24806	Excalibur_c29221_311	2B	140.4
650	28692	IWB28692	Excalibur_c73791_296	2B	140.4
651	59762	IWB59762	RAC875_c63343_52	2B	140.4
652	44889	IWB44889	Kukri_c39136_675	2B	140.4
653	38972	IWB38972	Ku_c25908_277	2B/6B	140.4
654	19318	IWB19318	D_GDS7LZN01D8KK0_75	2B	140.4
655	23770	IWB23770	Excalibur_c22201_907	2B	140.4
656	21063	IWB21063	Ex_c66300_924	2B	140.4
657	57127	IWB57127	RAC875_c35873_1894	2B	140.4
658	28533	IWB28533	Excalibur_c6937_1065	2B	140.4
659	22131	IWB22131	Excalibur_c12675_1147	2B	140.4
660	46023	IWB46023	Kukri_c49784_86	2B	140.4
661	46022	IWB46022	Kukri_c49784_56	2B	140.4
662	15636	IWB15636	D_contig12069_831	2B	140.4
663	8699	IWB8699	BS00056645_51	2B	140.4
664	8698	IWB8698	BS00056642_51	2B	140.4
665	65021	IWB65021	RFL_Contig5495_682	2B	140.4
666	65019	IWB65019	RFL_Contig5495_464	2B	140.4
667	48530	IWB48530	Kukri_c9898_1618	2B	140.4
668	13362	IWB13362	CAP12_c3254_366	2B	140.4
669	10624	IWB10624	BS00072840_51	2B	140.4
670	27000	IWB27000	Excalibur_c48871_625	2B	140.4
671	74748	IWB74748	tplb0042o21_419	2B	140.4
672	50794	IWB50794	Ra_c105904_187	2B	140.4
673	50793	IWB50793	Ra_c105904_1191	2B	140.4
674	26914	IWB26914	Excalibur_c47996_509	2B	140.4
675	41613	IWB41613	Kukri_c16621_417	2B	140.4
676	29266	IWB29266	Excalibur_c8919_345	2B	140.4
677	13403	IWB13403	CAP12_c449_277	2B	140.4
678	166	IWB166	BobWhite_c1105_745	2B	140.4

679	63909	IWB63909	RFL_Contig2324_884	2B	140.4
680	79867	IWA6164	wsnp_JD_c7305_8404286	2B	140.4
681	54767	IWB54767	RAC875_c19042_443	2B	140.4
682	48199	IWB48199	Kukri_c900_1334	2B	140.4
683	30139	IWB30139	Excalibur_rep_c105614_172	2B	140.4
684	9733	IWB9733	BS00066148_51	2B	140.4
685	75990	IWA988	wsnp_CAP12_c80_46551	2B	140.4
686	74736	IWB74736	tplb0042a21_1091	2B	140.4
687	63563	IWB63563	RFL_Contig1115_407	2B	140.4
688	10623	IWB10623	BS00072839_51	2B	140.4
689	77695	IWA3315	wsnp_Ex_c31064_39902843	2B	140.4
690	81209	IWA8018	wsnp_Ra_c58860_60407020	2B	140.4
691	32375	IWB32375	GENE-1304_735	2B	140.4
692	35082	IWB35082	IAAV5743	2B	140.4
693	23590	IWB23590	Excalibur_c21117_99	2B	140.4
694	12117	IWB12117	BS00100117_51	2B	140.4
695	80345	IWA6852	wsnp_Ku_c28820_38731137	2B	140.4
696	758	IWB758	BobWhite_c1523_269	2B	140.4
697	56745	IWB56745	RAC875_c3259_276	2B	140.4
698	50358	IWB50358	Kukri_rep_c78353_161	2B	140.4
699	45069	IWB45069	Kukri_c408_154	2B	140.4
700	42373	IWB42373	Kukri_c20793_249	2B	140.4
701	41512	IWB41512	Kukri_c16035_685	2B	140.4
702	32031	IWB32031	GENE-0676_715	2B	140.4
703	32030	IWB32030	GENE-0676_649	2B	140.4
704	28681	IWB28681	Excalibur_c7366_1926	2B	140.4
705	22135	IWB22135	Excalibur_c12675_1789	2B	140.4
706	48653	IWB48653	Kukri_rep_c101484_438	2B	140.4
707	30221	IWB30221	Excalibur_rep_c106698_235	2B	140.4
708	76440	IWA1667	wsnp_Ex_c12675_20144479	2B	140.4
709	8650	IWB8650	BS00054751_51	2B	140.4
710	77407	IWA2946	wsnp_Ex_c25043_34305764	2B	140.4
711	77133	IWA2551	wsnp_Ex_c2066_3877373	2B	140.4
712	70683	IWB70683	Tdurum_contig41912_402	2B	140.4
713	24252	IWB24252	Excalibur_c25043_1020	2B	140.4
714	11314	IWB11314	BS00083626_51	2B	140.4

715	7605	IWB7605	BS00026032_51	2B	140.4
716	76786	IWA2094	wsnp_Ex_c16074_24502385	2B	140.4
717	79557	IWA5694	wsnp_Ex_rep_c70525_69448648	2B	140.4
718	69974	IWB69974	Tdurum_contig30201_63	2B	140.4
719	1124	IWB1124	BobWhite_c18071_171	2B	140.4
720	22132	IWB22132	Excalibur_c12675_1395	2B	140.4
721	36702	IWB36702	Jagger_c6714_68	2B/2D	143.7

Supplementary table 6: Comparison of consensus map locations of SNP markers located in the *Sr7*-region from three different mapping studies

90K SNP ID	Rescaled 90K Consensus map distance (cM)	KASP/STARP markers[a]	Reference/ Source
IWB47901	137.9	-[b]	Current study
IWB24693	145.3	-	Current study
IWB71467	136.3	*Xrwgsnp10*	Saini et al. 2018
IWB73323	137.0	*Xrwgsnp11/kwh703*	Saini et al. 2018
IWA1067	136.8	-	Turner et al. 2016

[a]KASP- Kompetitive allele specific polymerase chain reaction (PCR) markers, STARP-SNP-based semi-thermal asymmetric reverse PCR markers
[b]No KASP marker was developed

Supplementary table 7:. Kompetitive allele specific (KASP) primer name, SNP name, chromosome location, and primer sequence information for markers in the *Sr7* (2BL), *Sr8a* (6A), and *Sr9* (2BL) chromosome regions

Primer name	SNP ID	Chr.[c]	Primer A1 (Tail1 at 5′-end)[d]	Primer A2 (Tail 2 at 5′-end)[e]	Primer C
kwh28	IWA7659	2BL	GTGACACCACTGAACCATAGGCA	GACACCACTGAACCATAGGCC	GTGATGTTGGATGGTGGCAAAAGGAT
kwh29	IWA2215	2BL	ACCCATCCCTACTAACAAACGACAA	CCATCCCTACTAACAAACGACAC	GTGACCTTGGGGTCACTGAAGATTT
kwh32	IWA3176	2BL	TGATCAGCATTGTCAGACTTGGCA	GATCAGCATTGTCAGACTTGGCG	TAATCTGTCAAAGGATTATAAGCATGCGAA
kwh53[a]	IWB5029	6A	AAGCTCCGGCTCTACTCCATC	CAAGCTCCGGCTCTACTCCATT	AGTCCCCCAGCGCGCTGCT
kwh54[a]	IWB22036	6A	ATTCGCTCAGTATCTGTTCTTCTTG	CTATTCGCTCAGTATCTGTTCTTCTTA	ATTGGAGACAGTTGCAGCCAGTGTT
kwh58[a]	IWB26414	6A	GTGCCGCCGAGCATCCTG	GGTGCCGCCGAGCATCCTA	GAAGATTCATCCTCTCCAGGCCAA
kwh62[a]	IWB72429	6A	CATCCGTAAGGTTAAATAAACTATCTACAA	ATCCGTAAGGTTAAATAAACTATCTACAC	CTGAAGCAGATGTTCTACTCTCAGGAA
kwh63[a]	IWB64918	6A	GCACTTGCGACTCGAGGGTT	GCACTTGCGACTCGAGGGTC	GGCCCGGAATCCGCCACCAT
kwh221[a]	IWB9013.2	6A	CGTCCGAACAAATTCAATGGAAAATTGT	GTCCGAACAAATTCAATGGAAAATTGC	TCACCCATGCTGAGAGGCAACAAT
kwh222[a]	IWB50596	6A	ACAAGAAGCAGCTTGCAATTCTCATAT	CAAGAAGCAGCTTGCAATTCTCATAC	TTTGTAACTGACAGTGAAAAAATGTCAGTT
kwh223[a]	IWA705	6A	GTGGACGCATCAATCTGTGGAC	CGTGGACGCATCAATCTGTGGAT	GAACAAATACGATGGACAGACACGTATAA
kwh225[a]	IWB28338	6A	GAGTTATCTTGAAGAGGCCTG	CCTGAGTTATCTTGAAGAGGCCTA	TATCCAATCACTGATCTGCTACAGATGAT
kwh227[a]	IWB42506.1	6A	GGCGGAGCGACGACTCACA	GCGGAGCGACGACTCACG	ACAATGTCGACACGAGCCTCAGAAA
kwh228[a]	IWB34187.1	6A	AGCAAACCCTAACCCTAGCCTG	CAGCAAACCCTAACCCTAGCCTT	ATCGGTCGACGGAGAAGCCGAT
kwh231[a]	IWB2817	6A	GAAATTAAAGTGGGTGCTTTGGAGCT	AAATTAAAGTGGGTGCTTTGGAGCG	CCGAGGCAAAGACGAATACACGTTT
kwh232[a]	IWB36255	6A	AGACCAAGAACTAATGTTGCTG	GGCTAGACCAAGAACTAATGTTGCTA	GACTGTTGGCCATTTACCGCTCTAA
kwh233[a]	IWB40518	6A	AGCAAGTATCTTGAGCGATGCAACA	CAAGTATCTTGAGCGATGCAACG	TTCAGCTTTTCGGTCTCACCTCTGA
kwh570	IWB45296	2BL	CAGGAGCTTGGATGAGGCTACAT	AGGAGCTTGGATGAGGCTACAC	TGGAGGCTACAACGCTATACTGCAT
kwh572	IWA6453	2BL	CCAGCCATTCTGAAGGTGGAA	CCAGCCATTCTGAAGGTGGAG	TCCTTCGGTCGCACTTTTCT
kwh574	IWA6076	2BL	GCGAAACAAAATCTGAAGCTATTCT	GCGAAACAAAATCTGAAGCTATTCC	GCAAAAAGCTCTGATGTACCG
kwh575	IWB11092	2BL	ACAGATGAAGCGTTCTGTTACA	ACAGATGAAGCGTTCTGTTACC	CCCCGACACAACCATGGTAA
kwh576	IWB28337	2BL	AGAGTCACACAACTGAAGATAGTAA	AGAGTCACACAACTGAAGATAGTAG	TGATCATCCTCAAACAGTGTATCA
kwh577	IWB51068	2BL	AGGTTTTCTACTTCTGTCTCGAT	AGGTTTTCTACTTCTGTCTCGAC	CCTCCATGAAAGAGGTCGCT
kwh578	IWB36072	2BL	CCACCACACCCTCACCGA	CCACCACACCCTCACCGG	CTTAGGCCGAGGATTTGTCAT
kwh579	IWB10846	2BL	CGAGTCCTATGAGAGCCATGAA	CGAGTCCTATGAGAGCCATGAG	TGAACCACTGCTCACCACTT
kwh580	IWB26300	2BL	GTCGAATACGGGGCAGCAA	GTCGAATACGGGGCAGCAG	GAGAAATCGACTCACCGGCT
kwh583	IWB12118	2BL	CCCCAAGGCCTCTTTCGT	CCCCAAGGCCTCTTTCGG	GCCAGTTTGATGTCGAAGAGAT
kwh585	IWA3252	2BL	GAGCCACTGATCTGATCACTT	GAGCCACTGATCTGATCACTC	TCGTCGGTGTTTCCCTGTTT
kwh586	IWB10173	2BL	CAAAACAGGGCTGCTGCAAT	CAAAACAGGGCTGCTGCAAC	CTCCTGTGTGGCAGCTATGA
kwh587	IWB40455	2BL	CTGACCAGTTTCAATGTACCAAAT	CTGACCAGTTTCAATGTACCAAAG	TACACTGCAGTCCTGGGGT
kwh588	IWA1305.1	2BL	CATTACACAAAGAGGAGCAAGTTT	CATTACACAAAGAGGAGCAAGTTC	TCCGTCAGTTGCTTCCATGT
kwh589	IWB36041	2BL	CAAGGACCTACTGAAAGA	GCTCAAGGACCTACTGAAAGG	GTAGCTCGTTGACCACCTCA
kwh590	IWB64817	2BL	AGTAGCATTTGGCCATGATTCA	AGTAGCATTTGGCCATGATTCG	GCAGGATAAGGTGCCTTCA
kwh592	IWB36708	2BL	CCAATCAAGCCGGAGTACA	CCAATCAAGCCGGAGTACG	TGATCAGATCACATATGAGATGGAT

kwh593	IWB12643.1	2BL	ATGGGGTGGTTCTCTAATTTCAT	ATGGGGTGGTTCTCTAATTTCAC	TGGGAGTACTTCTCATATTACTCG
kwh596	IWB56961	2BL	CATTCCATTCTATCAAATTGCCGT	CATTCCATTCTATCAAATTGCCGC	CACTGCCGACACTTGCATAG
kwh598	IWB8280	2BL	TGACTTTAACTTTATTGGTACCCTT	TGACTTTAACTTTATTGGTACCCTG	CCGCAATCCATATGTGCGAC
kwh599	IWB10434	2BL	TGATTGCGGAGTATGTGCA	TGATTGCGGAGTATGTGCG	GCAATGCGTGTCCGTGTAAATA
kwh696	IWB19553	4AL	CCATAACTGAGACGACGAACTCT	CCATAACTGAGACGACGAACTCG	CCCGTTTCCTTGGAGTAATGCCAAA
kwh697	IWB52406	4AL	GACCTGCAAAATAGCTCAGTGAAGA	ACCTGCAAAATAGCTCAGTGAAGG	TACCCAGTTCCCTCAGATTGGTCAA
kwh698	IWB12229	4AL	GAAGTCTACCCTTGAATACAAATCATAATTAA	AGTCTACCCTTGAATACAAATCATAATTAG	CGTACAACAATATGCATAATACGTATAGAT
kwh699	IWA1674	4AL	AACTCAAAAGGATGTGAAGGTTTGTCA	CTCAAAAGGATGTGAAGGTTTGTCG	GAAGCAACAAACCAGTGAAGCTTCCTT
kwh703[b]	IWB73323	4AL	GTCAGTTTCAGATGAAAAGGCCAGT	CAGTTTCAGATGAAAAGGCCAGC	AACTCATTTGATGGTTTCTGTATAATGGAT

[a and b]Refrences- Hiebert et al . 2017and Saini et al. 2018; [c]Chromosome name;
[d]Tail-1 has a 5′- FAM tail- GAAGGTGACCAAGTTCATGCT and [e]Tail-2 has a VIC tail- GAAGGTCGGAGTCAACGGATT
